# Supplementary material for: Convergent genomic signatures of domestication in sheep and goats
Source: Nat Commun. 2018 Mar 6;9:813. doi: 10.1038/s41467-018-03206-y (PMC5840369; doi:10.1038/s41467-018-03206-y)
Supplement: Supplementary file 1 — Supplementary Information [file 41467_2018_3206_MOESM1_ESM.pdf]

Convergent genomic signatures of domestication in sheep and goats

Alberto et al.

Supplementary Notes

• **Supplementary Note 1: Genome diversity**

Variant discovery and calling

A total number of 33,395,376 polymorphic SNPs were discovered and successfully called on the 73 *Ovis* samples. Comparatively, 22,969,973 SNPs were discovered and called on the 72 *Capra* samples. Related to the genome sizes, these numbers corresponded to densities of one SNP every 74bp and 105bp in *Ovis* and *Capra,* respectively.

Cross-genus alignments

The cross alignments between the two reference genomes produced a high coverage of the non-reference genome (above 90%), which is almost as high as the percentage covered for the reference genome itself (Supplementary Table 6). In *Ovis*, we found that 23,839,165 SNPs could be placed on the goat reference unambiguously (71.7% of discovered sites). Similarly, 16,359,479 SNPs in *Capra* could be placed on the sheep reference unambiguously (71.2% of discovered sites).

• **Supplementary Note 2: Demographic inferences**

The demographic inferences of the *Ovis* and *Capra* populations (Supplementary Fig. 2) show similar variations in both genera, in particular between 1 million years ago (ya) and ~30,000 ya. For both genera the MSMC analysis suggests an initial effective population size (Ne) decline between 1 million ya and 500 kya to 300 kya, which was followed by an expansion reaching a maximum Ne at ~170 kya. At this time *Ovis* presented a relatively larger Ne than *Capra*. This initial expansion was followed by a second decrease that ended around 50,000 ya prior the start of the last glacial period. Following this period both genera started recovering demographically. In *Capra* the demographic variations are heterogeneous

for both the ancestors of wild and domestic modern populations since about 30 kya. This is in line with the contribution of an assembly of different ancestral lineages to the modern domestic stocks and wild populations, as already shown by the study mitochondrial haplogroups[1]. In *Ovis* the variations of Ne were similar in the ancestors of both wild and domestic populations. From the period of domestication (~10,500 ya), two distinct demographic histories are inferred for the bezoar ibex (corresponding to different genetic subgroups of IRCA represented in blue in Supplementary Fig. 7b) with one of the lineages being quite stable while the other carried on expanding demographically until recently. For the goats lineage, the Ne shows a marked decline until the recent past. A similar pattern is observed for *Ovis*, where the mouflon started to present a different demographic history with respect to sheep near the onset of domestication. However, contrary to the bezoar ibex, mouflon seem to have carried on expanding until the recent past. Sheep, similarly to goats, achieved a dramatically Ne decline.

MSMC does not have sufficient resolution for very recent time points preventing us from saying much about the demographic history of these species over the last 1,000 years. For the earliest period (i.e., before 300 kya), the ancestral Ne of *Capra* appears larger than that of *Ovis*, but current methods prevent us from seeing further back in time and better characterise that period. We must also caveat the fact that the mutation rate used is not goat/sheep specific, what might affect the interpretation of the timing (see Methods).

• Supplementary note 3 : Genetic structure

Genetic diversity within groups

Among *Ovis* groups, the IROO group displayed 24.9 million polymorphic SNPs, while the IROA, MOOA and wpOA groups had respectively 20.7, 21.4 and 21 million polymorphic SNPs (Supplementary Table 1). Although the IROO group presented the lowest sample size with 13 individuals, it also showed the highest level of mean nucleotide diversity (π=2.68x10^{-3} against π=2.20x10^{-3} on average for the three domestic groups). Individual inbreeding values varied largely within groups (Supplementary Data 5), however the average inbreeding

coefficient in the IROO group (F=0.08) was significantly lower (p-value<10^{-4}) than in the wpOA group (F=0.18), while IROA and MOOA showed intermediate values (Supplementary Fig. 1). The average relatedness was 0.859 for the 60 sheep and 0.823 for the Asiatic mouflon.

Among *Capra* groups, IRCH, showed the highest number of polymorphic SNPs (15.4 million). MOCH showed 13.1 million SNPs polymorphic, while IRCA and wpCH had respectively 11.3 and 10.4 million SNPs polymorphic (Supplementary Table 1). Nucleotide diversity was higher for IRCH (π=1.65x10^{-3}) than for IRCA (π=1.53x10^{-3}) wpCH (π=1.54x10^{-3}) and MOCH (π=1.47x10^{-3}). The wpCH and IRCA groups showed similar average inbreeding (F=0.22 for both), which was significantly higher than IRCH (F=0.08), while it was intermediate for MOCH (F=0.15) (Supplementary Fig. 1). The average relatedness was around 0.915 between the 54 domestic goats, and 0.916 between Bezoar ibex (Supplementary Data 5).

Genetic structure among groups

The weighted whole-genome *Fst* index showed an overall level of genetic differentiation among all *Ovis* groups of 0.050. The highest values were obtained for pairwise comparisons including the wild group IROO and one of the domestic groups (*Fst* = 0.075 on average). On the other hand the average pairwise *Fst* value among the IROA, MOOA and wpOA groups was only 0.013. For *Capra*, the overall *Fst* was 0.047 considering the four groups together. The average pairwise *Fst* value between the three domestic groups IRCH, MOCH and wpCH was 0.030. For pairwise comparisons between each domestic group and the wild group IRCA, Fst was 0.048 with IRCH and 0.065 both with MOCH and wpCH.

For the following analyses of genetic structure and admixture we removed the SNPs showing high LD (see Methods), keeping 6,155,224 and 6,511,536 SNPs for *Ovis* and *Capra*, respectively. Using sNMF[2] we estimated the number of genetic clusters in the data and the admixture between them. The best partition of samples into clusters for each genus was estimated with the cross-entropy criterion resulting in K=2 for *Ovis* and K=3 for *Capra*.

Nevertheless, we further investigated the effect of higher values of K to assess how the partition of individuals changed for different levels of the genetic structure. For both *Ovis* and *Capra*, the clustering analysis first separated the wild animals from the domestics. For K=2, the domestic and the wild *Ovis* animals belonged clearly to two different clusters. For K=3, the domestics were split in different clusters according to their geographic origins, with the wpOA individuals being assigned to the cluster representing either IROA or MOOA or being admixed between both. For K=4 and K=6 two inbred and one other IROO individuals were clustered apart, while at K=5 a new cluster appeared with various levels of admixture in the wpOA group (Supplementary Fig. 7a). For *Capra*, the clustering for K=2 showed one cluster representing the domestic animals, while the second cluster regrouped 8 IRCA animals and the remaining 12 animals were admixed. At K=3, the European goat breeds belonged to a distinct cluster, and at K=4 the Iranian and the Moroccan goats were clustered separately. Then, for K=5 and K=6 the admixed IRCA individuals were largely assigned to two new clusters of respectively 7 and 3 individuals (Supplementary Fig. 7b). Notably, when increasing the number of clusters we found no admixture between wild and domestic animals for both *Ovis* and *Capra*.

Tests for admixture

As the genetic clustering clearly separated wild and domestic individuals, we rooted the TreeMix tree by the split between both lineages. In sheep, most of the wpOA individuals were more closely-related to MOOA than IROA individuals, that clearly clustered separately (Supplementary Fig. 3a). In goats, the IRCH and MOCH groups were also clearly separated, and while the European breeds from wpCH were close to the MOCH group, the Australian breeds clustered with IRCH (Supplementary Fig. 3b). Further post-processing with the dedicated TreeMix R package[3] confirmed that the topologies recovered in absence of migration edges explained nearly all differences in the allelic frequencies among groups (99.1% and 98.2 of the total variance for *Ovis* and *Capra*, respectively, Supplementary Table 3). Moreover the residual values were high only for intra-species pairs of individuals (i.e., not

between wilds and domestics, see residuals heat maps in Supplementary Figure 3). We also explored the possibility of minor admixture events, adding an increasing number of migration edges, from 1 to 4 (Supplementary Table 3). All the migration edges involved individuals from the same species (no wild-domestic migration). For *Ovis* no sensible increment of the variance was observed. The maximum increment of the variance in *Capra* (>99%) was reached by adding a single migration edge between domestics, which was inferred from one Moroccan individual to a European breed. A formal test of admixture based on the *f3* statistics[4], however, showed no significant results for any combination of groups (Z-scores > 0, Supplementary Table 6), providing no evidence for any recent admixture among the wild and domestic species within each genus. It is worth noting that this finding does not reject ancient introgressions of genetic material, as domestication and intensive selective breeding may have eroded the molecular signature of admixture.

• Supplementary Note 4: Detection of selection signatures

The subsets of variants filtered on allelic frequencies (see Methods) were 22,134,330 for the 53 *Ovis* samples and 12,412,758 for the 58 *Capra* samples used in this analysis.

hapFLK results

We filtered out from hapFLK results the genomic regions for which the haplotype clustering was not congruent among the domestics. Thus, we retained a total of 8,498 SNPs for *Ovis* and 10,571 SNPs for *Capra* with q-values<10^{-2} from the FDR framework applied to the whole set of variants. Merging these SNPs, we obtained respectively 30 and 44 genomic regions in *Ovis* and *Capra*, respectively, among which 3 pairs of regions were homologous between both genera (Supplementary Table 4 and Supplementary Data 3).

Detection of homologous regions under selection

Before running the stratified FDR analysis, we first removed one region detected by hapFLK in *Ovis* due to repeated sequences that affected the quality of the cross-alignment with

Capra. We then applied the stratified FDR approach, analysing separately for each genus the regions homologous to those detected as significant in the other genus on one hand (shared stratum), and the rest of the genome on the other hand (general stratum).

Applying the stratified FDR to the shared stratum, we detected among the segments homologous to the 44 regions previously detected in *Capra* 12,016 SNPs corresponding to 19 genomic regions in *Ovis* (q-values<10^{-2}). In *Capra*, 1,698 SNPs located on 4 regions were detected. These regions contained 3 homologous regions previously detected without stratification. When applying the stratified FDR method to the general stratum, we detected 7,091 SNPs and 9,121 SNPs, which were located in 27 and 40 regions in *Ovis* and *Capra*, respectively (Supplementary Table 4). These regions were identical to the non-homologous regions detected without stratification, minus one that was not significant anymore in *Capra*.

Thus, the stratified FDR approach globally detected 46 genomic regions including 18,556 SNPs as genomic signatures related to domestication in *Ovis*. Their size varied between 2.4kb to 254kb and they represented a cumulated length of 3.402Mb, which is equivalent to 0.13% of the total autosome length. According to gene annotations, 9 regions were intergenic and 37 contained a total of 145 genes, which represents 0.6% of the genes annotated in sheep. In *Capra*, a total of 10,819 SNPs located in 44 genomic regions were detected as signatures related to domestication. Their size ranged between 0.1kb to 278kb for a cumulated length of 2.811Mb, which represented 0.11% of the autosomes. While 8 regions were intergenic, the 37 remaining regions contained 147 genes, *i.e.* 0.7% of the total number of genes annotated in goat.

Based on the cross alignments between the sheep and goat reference genomes, we found 20 pairs of regions to be homologous between genera (Supplementary Fig. 8). Using the coordinates of SNPs on the two reference genomes we found that the signals were overlapping in 13 cases.

Assessment of the patterns of selection

Based on the difference in nucleotide diversity ($\Delta\pi$) between wilds and domestics in the

detected regions, we identified 45 regions with a lower diversity in sheep than in mouflon (i.e., stabilizing and/or directional positive selection) and one region with a higher diversity in domestics (i.e., relaxation or diversifying selection in the domestics, or recent positive selection in the wilds). For *Capra*, 27 regions displayed a lower diversity in goats (e.g., positive selection) and 17 a higher diversity corresponding to other patterns of selection. Among the homologous regions, the pattern of selection was concordant in both sheep and goat for 16 regions and different for 6 regions (Supplementary Data 3).

Functional interpretations

Among the genes found in the genomic regions under selection, we kept the closest to the SNP with the lowest q-value (*i.e.* top signal) as the most likely gene targeted by selection. In addition to 3 genes with unknown functions, we found a total of 59 genes that were involved in several GO child terms of Biological Processes (Supplementary Data 4). The distribution of these 59 genes in the GO categories differed from that of the reference distribution (i.e., the 18,689 human genes associated to GO terms in Swiss-Prot) due to an excess of genes related to pigmentation and, to a lesser extent, in biological adhesion and rhythmic process (Chi-square test, p-value≤0.05, Supplementary Data 4). The overrepresentation of genes involved in pigmentation might reflect the importance of coat colour as a target of selection in livestock[e.g., 5]. Furthermore, most of the 59 genes found under selection have already been associated to phenotypic effects in livestock species. We found 14 genes related to immunity, 25 genes to different productivity traits such as milk (11 genes), meat (11 genes), fertility (2 genes) and/or hair (4 genes) characteristics, and 5 genes related to the neural development or nervous system (Supplementary Data 3). The other genes mostly corresponded to general functions without identified phenotypic effect in livestock species, or to uncharacterised functions. We classified these genes in an "Other " category in Table 1 and Supplementary Data 3, and their related GO terms are available in Supplementary Data 4. The representation of the GO terms did not differ between the genes from homologous regions and the whole set of genes under selection (Chi-square test, p-value>0.9,

Supplementary Data 4). In 10 cases where a gene was found in the homologous regions, the genes most likely impacted by selection were the same between *Ovis* and *Capra* (Table 1). In 4 other cases the genes were different but involved in similar phenotypic effect in livestock, while the genes from 3 regions corresponded to different classes. Three regions were intergenic in both *Capra* and *Ovis*. Four genes common to sheep and goat (*HMGI-C*, *KITLG, MTMR7,* and *NBEA*) show clear pleiotropic effects. *HMGI-C* is involved in body size in sheep[6] and was also described as being responsible of dwarf size in chicken[7]. *MTMR7* is expressed in the central nervous system in human[8] and is also involved in fatty acid composition in pig[9]. *KITLG* is known to have an effect on coat coloration in numerous mammal species[10], is associated to litter size in goat[11] and is also implicated in nerve cells development and mast cells development, migration and function[12]. *NBEA* is associated to wool crimp in sheep[13] but is also known to regulate neurotransmitter receptor trafficking to synapses in human[14] and was suspected to play a role in docility in cattle[15].

In 21 regions among the 46 detected with hapFLK in *Ovis*, 148 SNPs showed combined FLK p-values<10^{-4}. According to VEP, they were mostly located in intergenic regions (92 SNPs) and intronic sequences (44 SNPs), whereas 12 SNPs were upstream or downstream genes and two SNPs were exonic (one missense and one synonymous, see Supplementary Table 5). This distribution was not significantly different from that of the whole set of SNPs initially tested. In *Capra*, 928 SNPs located in 29 out of the 44 regions detected with hapFLK showed combined FLK p-values<10^{-4}. They were found in intronic sequences (544 SNPs), in intergenic regions (296 SNPs), upstream or downstream genes (190 SNPs). Within coding sequences, only one missense variant was found. There was here a clear enrichment for variants located in non-coding regions close to genes (intronic, upstream gene and downstream gene) compared to the whole set of SNPs analysed (Chi-square test; p-value<$2x10^{-16}$; see Supplementary Table 5).

• Supplementary Note 5 : Comparison between selection detection methods

Following a reviewer's comment for exploring and discussing the differences between our approach and that of Naval-Sanchez et al. (2018)[16] who studied a sampling including some identical individuals, we provide the following information about the impact of the method and sampling on the detection of signatures of selection.

Our study was specifically designed to detect selected sweeps in common between sheep and goat. This stems from both the sampling (ie. working on traditionally-managed domestics with the same origin and wild populations from the cradle of domestication) and the choice of the method (ie. hapFLK with stratified FDR approach). Naval Sanchez et al.[16] used an other approach based on Fst and pi (nucleotide diversity variation) to contrast the genomes of wild mouflon to that of a worldwide panel of 43 breeds (each represented by only a few individuals). This experimental design is very different from ours and designed to detect different selective processes. The method used by Naval-Sanchez et al. for detecting selective sweeps on 20 kb windows combined the mean Fst value and the difference in nucleotide diversity between wilds and domestics — estimated by ln(pi_wilds/pi_domestics). Combining the two variables make this method relaxed in a statistical sense. The thresholds applied for selecting the outlying windows (e.g., p-values on z-scores of 10^{-2} in Naval Sanchez et al.[16] does not reflect the true level of significance due to the non-independence of the two distributions that are combined. This relationship between Fst and ln(pi-ratio) can be seen on Supplementary Figure 9 (shape of the scatter-plot). The selection of outliers with the Fst/pi-ratio approach is thus very likely to be less stringent. hapFLK, however, is based on the difference in haplotype frequencies between populations and takes into account the hierarchical structure of the sampling. Its detection power has been proved to be greater than that of the Fst approach[6], is only slightly affected by migration and is not affected by bottlenecks. Even if Fst approaches are more commonly used for detecting selective sweeps, our study is much more suited to the hapFLK approach, even more when

considering our sampling made of populations with different drift effects.

Whatever this better theoretical support for the hapFLK approach, we analysed the same dataset with the two methods (HapFLK versus Fst/pi-ratio) in order to assess how the detection method would affect the result. We also assessed the effect of the sample set on the detection of selected genomic regions. As hapFLK is designed to detect selection among hierarchically structured populations, it cannot be applied to the worldwide panel, which are too heterogeneous as they are composed of breeds with very different demographic histories admixture, bottlenecks,...), and each breed is represented by a few individuals. Thus we applied the Fst/pi-ratio method on our sheep dataset and performed the following analyses:

- Effect of the method used. Reproducing the design used for the hapFLK analysis and using the method used by Naval-Sanchez *et al.*, Fst and pi ratios were calculated for 20kb windows between (i) Moroccan (MOOA) and wilds (IROO), (ii) Iranian (IROA) and wilds (IROO). Mean values were computed and outlying windows were detected using the combined z-transformed values with Bonferroni correction (Supplementary Figure 9). We then looked at the significant 20 kb windows which fell into the regions detected with hapFLK (green dots on Supplementary Figure 9). This represented 35 regions out of the 46 retained with hapFLK. On the 11 remaining regions, 6 were close (5-100 kb) to an outlying window with the Fst/pi method (represented by green stars on Supplementary Figure 10). Three regions found with hapFLK did not contain any 20kb windows with at least 20 SNP and were not analysed with the Fst/pi method to follow Naval-Sanchez *et al*. As a result 91% of the genomic regions (41/46) found with hapFLK in our study were also found with the Fst/pi method which, as expected, was more permissive (see above) with many other outlying windows (Supplementary Figure 9).

- Effect of the sampling. We also performed the Fst/pi analysis by contrasting to the wild group IROO either the whole worldwide panel (67 individuals used by Naval-Sanchez *et al.*) or our worldwide panel (wpOA: 20 individuals, subsample of the previous group). 41 % (798/1937) of the selective sweeps (for 20kb regions) detected were common to both

analyses. This shows the effect of subsampling in the world panel which is heterogeneous and composed of 43 different breeds. At the gene level (after merging 20kg regions closer than 50kb and looking at overlapping genes), this corresponded to an overlap of about 46 % (254/ 557, see Supplementary Figure 11).

To conclude, the Fst/pi method is not adequate to analyse our dataset, as the result might be affected especially by the differential effects of drift in the populations. However, it confirmed most of the sweeps detected with hapFLK but appeared to be more permissive. Moreover, the sweeps that we describe are confirmed by the haplotypes shown on Supplementary Figure 5. The genomic regions detected as under selection might depend on the composition of the worldwide panel, probably due to the different demographic and selective history of the component breeds. This justifies that we did not use the worldwide panel to detect selective sweeps in our study, but only a posteriori, to confirm the sweeps detected in traditionally managed populations.

• Supplementary Table 1 : Genetic statistics for the different groups of Ovis and Capra.

For each group the sample size, the number of segregating SNPs (S), the average nucleotide diversity (π), the mean inbreeding coefficient (F) and the mean genetic load over the whole genome are indicated. The p-value of the difference between the wild group and each domestic group is given in brackets (one-sided t-test for F and load, Mann-Withney test for π). For π, the Mann-Whitney tests between all pairs of groups for each genus all showed p-values < 2.2e-16. For mean inbreeding F and mean genetic load, the p-values of the one-sided t-tests on individual values between the wild group and each of the domestic group correspond to: ns: p-value>10-1 ; a: 5x10-2<p-value<10-1 ; b: 10-2<p-value<5x10-2 ; c: p-value<10-2 .

Ovis					
**Group**	**Sample size**	***S***	π (x10-3)	**Mean F**	**Mean load (x10^{-4})**
**All**	73	33,233,605	2.41	0.139	8.4
**IROO**	13	24,928,891	2.68	0.083	8.1
**IROA**	20	20,659,837	2.15	0.128 (a)	8.4 (a)
**MOOA**	20	21,351,476	2.22	0.139 (a)	8.5 (a)
**wpOA**	20	21,041,532	2.24	0.186 (c)	8.7 (b)

Capra					
**Group**	**Sample size**	***S***	π (x10-3)	**Mean F**	**Mean load (x10^{-4})**
**All**	72	22,969,973	1.70	0.163	4.7
**IRCA**	18	11 369 495	1.53	0.220	4.9
**IRCH**	20	15,425,601	1.65	0.084 (c)	4.5 (c)
**MOCH**	20	13,054,034	1.47	0.150 (c)	4.6 (c)
**wpCH**	14	10,384,091	1.54	0.219 (ns)	4.7 (c)

• Supplementary Table 2: Enrichment analysis for genes showing higher genetic load in sheep than in Asiatic mouflons.

Analysis performed with Webgestalt, showing significant results only for the category "phenotype" in Human.

**Model organism**	**Phenotype**	adjusted p value
Human	Cervical subluxation	0.002
Human	Hypoplasia of the odontoid process	0.002
Human	Abnormality of the odontoid process	0.003
Human	C1-C2 subluxation	0.01
Human	Hypertelorism	0.01
Human	Abnormality of the curvature of the vertebral column	0.01
Human	Aplasia/Hypoplasia of the nasal septum	0.01
Human	Abnormality of the ilium	0.01
Human	Scoliosis	0.01

• Supplementary Table 3: Part of the variance explained by the Treemix model according to the number of migration events.

# migrations	*Ovis*	*Capra*
0	0.9910066	0.9817221
1	0.9923272	0.9904986
2	0.9930432	0.9904883
3	0.9931297	0.9904724
4	0.9932956	0.990492

• Supplementary Table 4: Number of genomic regions detected with and without the stratified FDR approach

	Ovis		*Capra*		Homologous Regions
	SNPs	*Regions*	*SNPs*	*Regions*	
FDR whole genome	8,498	30	10,571	44	3
stratified FDR general stratum	7,091	27	9,121	40	0
stratified FDR shared stratum	11,465	19	1,698	4	20
**Total stratified FDR**	**18,556**	**46**	**10,819**	**44**	**20**

• Supplementary Table 5: Variant Effect Predictor categories of SNPs with FLK p-values<10^{-4} in *Ovis* and *Capra*.

The differences between the distributions of SNPs with FLK p-values<10^{-4} and all SNPs used for detecting selection signatures are tested with a Chi-square test.

		Intergenic	Intronic	Up/Down stream	Exonic	Chi square test
Ovis	FLK ($p<10^{-4}$)	92	44	12	2	p-value=0.3436
	All SNPs	14,560,617	6,139,562	1,697,583	125,442	
Capra	FLK ($p<10^{-4}$)	296	544	221	1	p-value < $2x10^{-16}$
	All SNPs	7,671,667	4,068,808	882,600	9,007	

• **Supplementary Table 6: Results of the pairwise alignment between the two reference genomes using a) sheep or b) goat as the reference species**.

Genomic regions of the reference species are forced to map uniquely to single loci of the non-reference species, whereas non-reference genomic regions are allowed to map to multiple locations of the reference species.

a)

Genome	Genome coverage in alignment	Total genome length	Percentage genome covered by alignment
Sheep	2,390,790,161	2,619,037,772	91.3%
Goat	2,357,251,044	2,607,448,494	90.4%

b)

Genome	Genome coverage in alignment	Total genome length	Percentage genome covered by alignment
Sheep	2,357,262,884	2,619,037,772	90.0%
Goat	2,388,436,682	2,607,448,494	91.6%

• **Supplementary Figure 1**

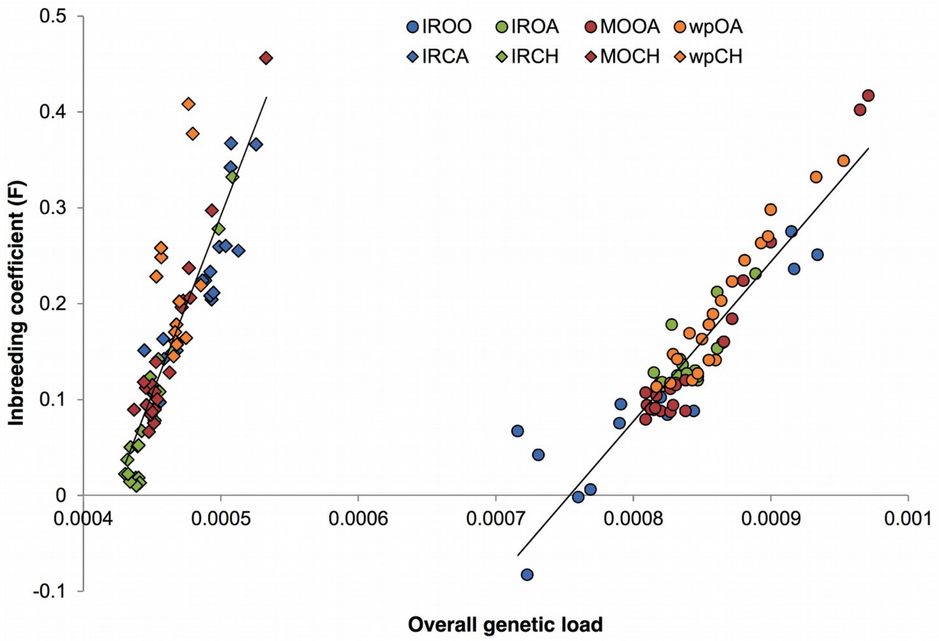

Supplementary Fig. 1: Correlation between inbreeding coefficient and genetic load.

Blue: wild, green: Iranian domestics, red: Moroccan domestics, orange: world panel.

• **Supplementary Figure 2**

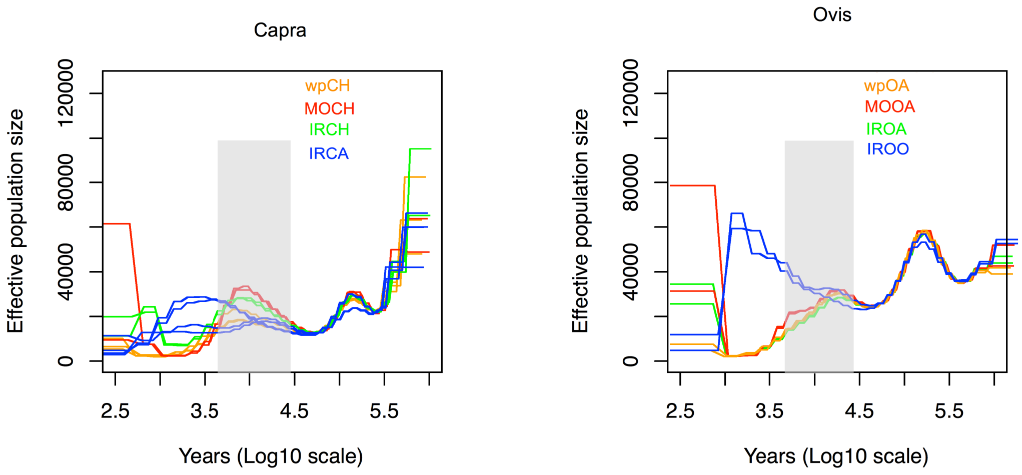

Supplementary Fig. 2: Time variations of the effective population size inferred with MSMC for *Capra* and *Ovis* groups.

Two samples of 2 different individuals were analysed for each group, except for IRCA where 4 samples were analysed to represent the genetic substructure of this group. Time scales were inferred with a mutation rate of 2.5 10^{-8} and a generation time of 2 years. The grey bar represents the rough period of domestication taking into account the uncertainty of time estimates.

• **Supplementary Figure 3**

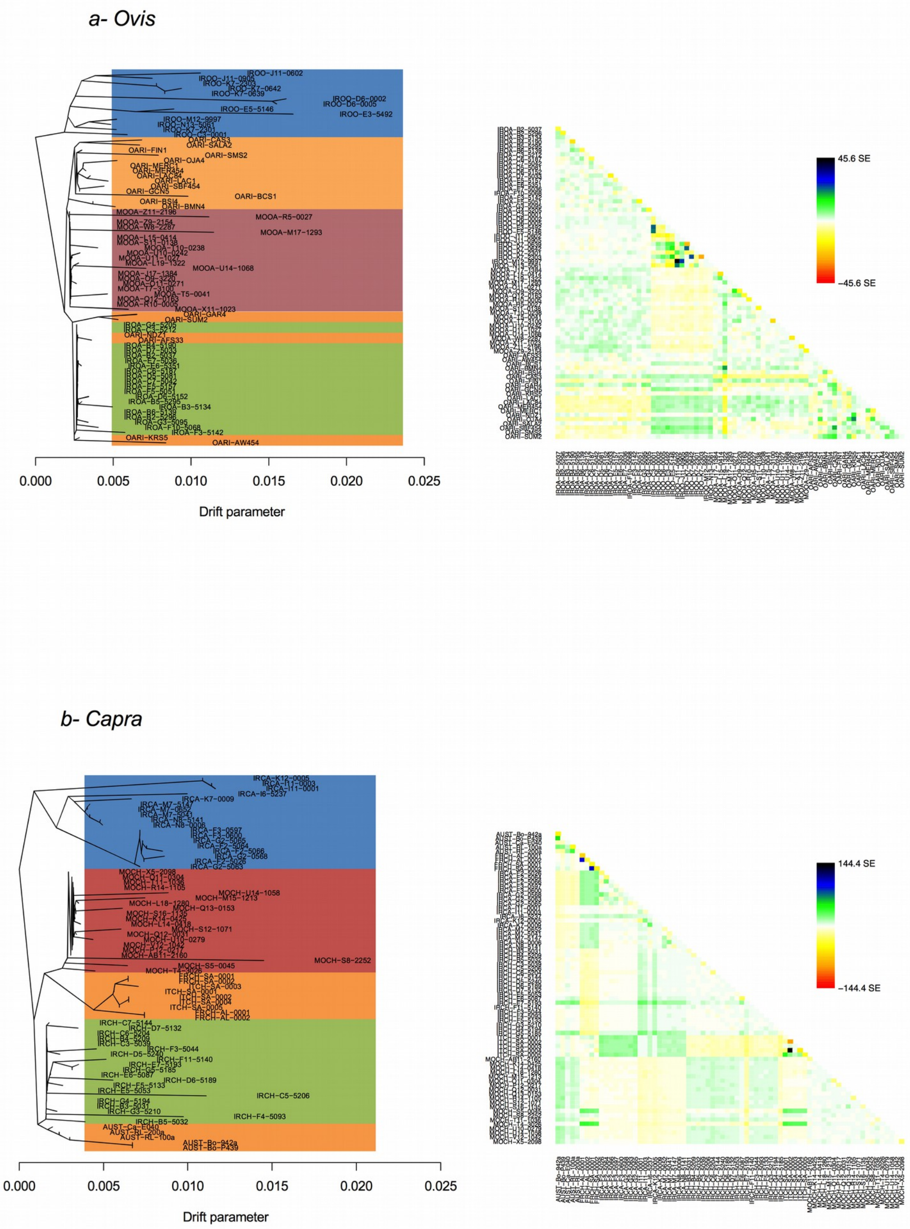

Supplementary Fig. 3: Maximum Likelihood trees showing genetic affinities among each genus in the TreeMix analysis, and the semi-matrix of residual values for 0 migration edges for *Ovis* and *Capra*.

• **Supplementary Figure 4**

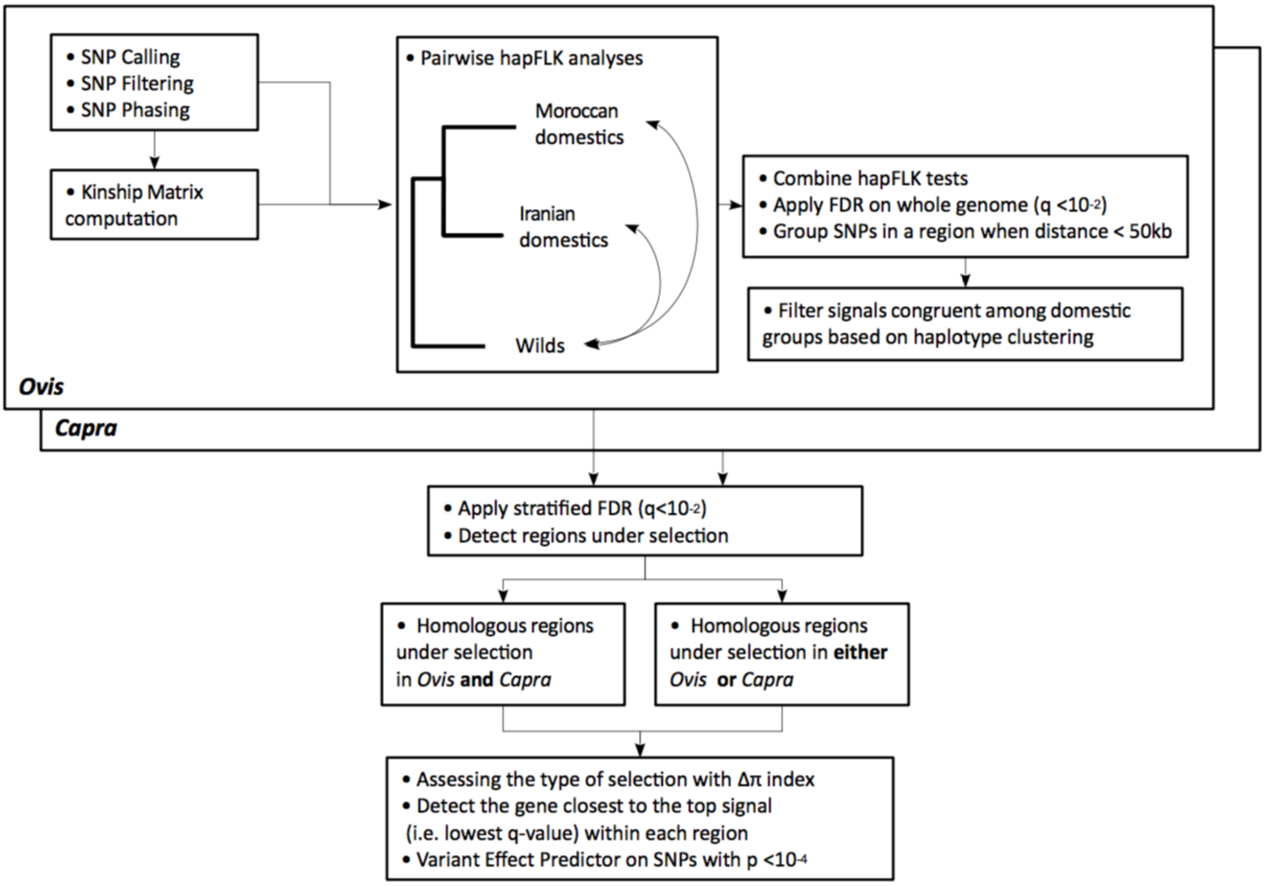

Supplementary Fig. 4: General strategy for detecting signatures of selection.

• Supplementary Fig. 5

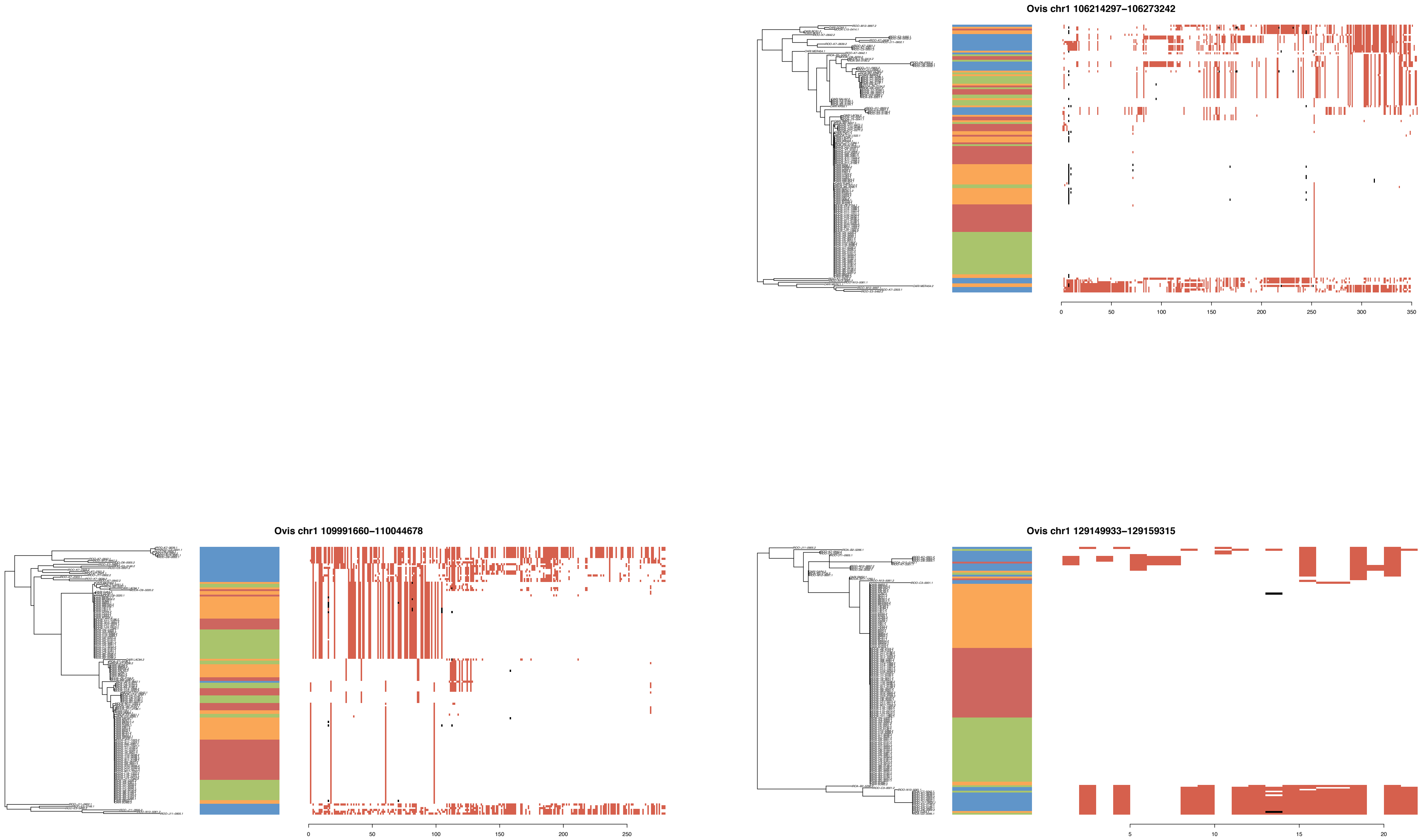

Supplementary Fig. 5 continued

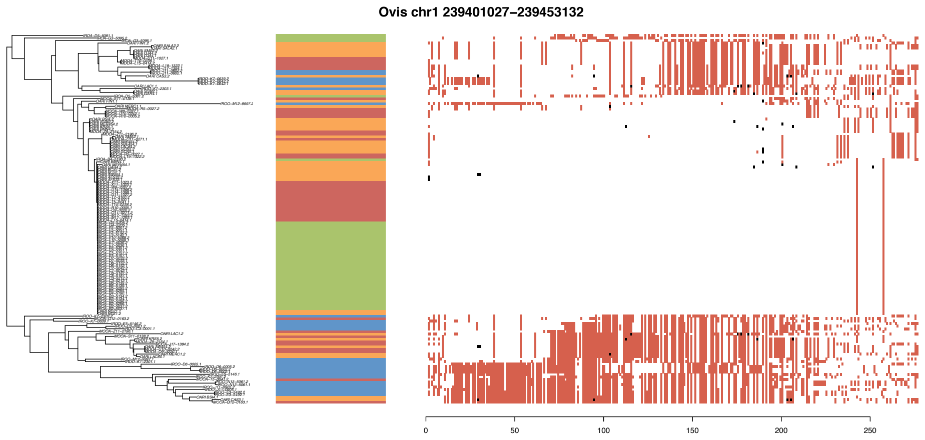

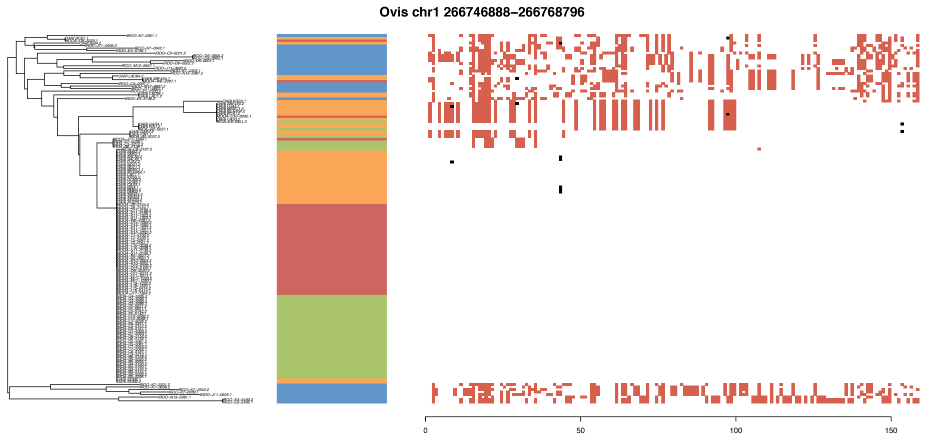

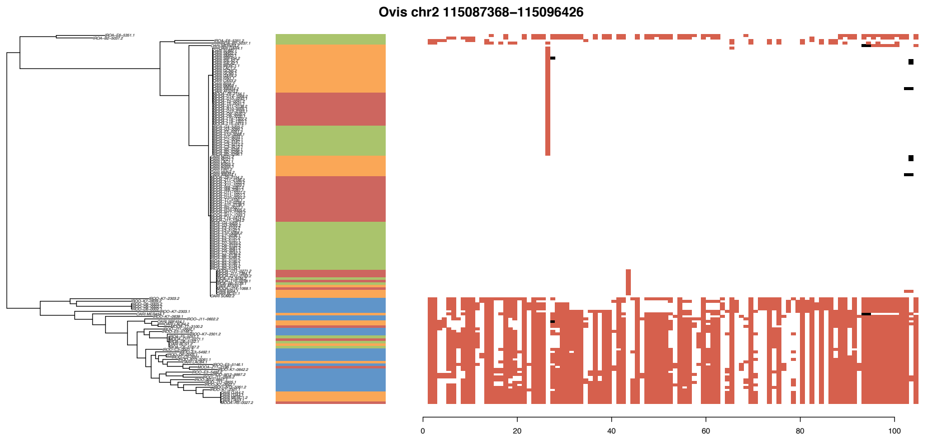

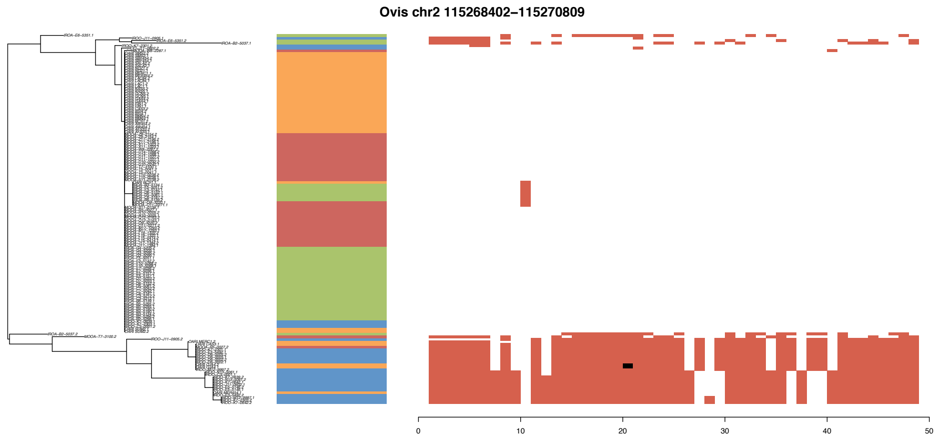

Supplementary Fig. 5 continued

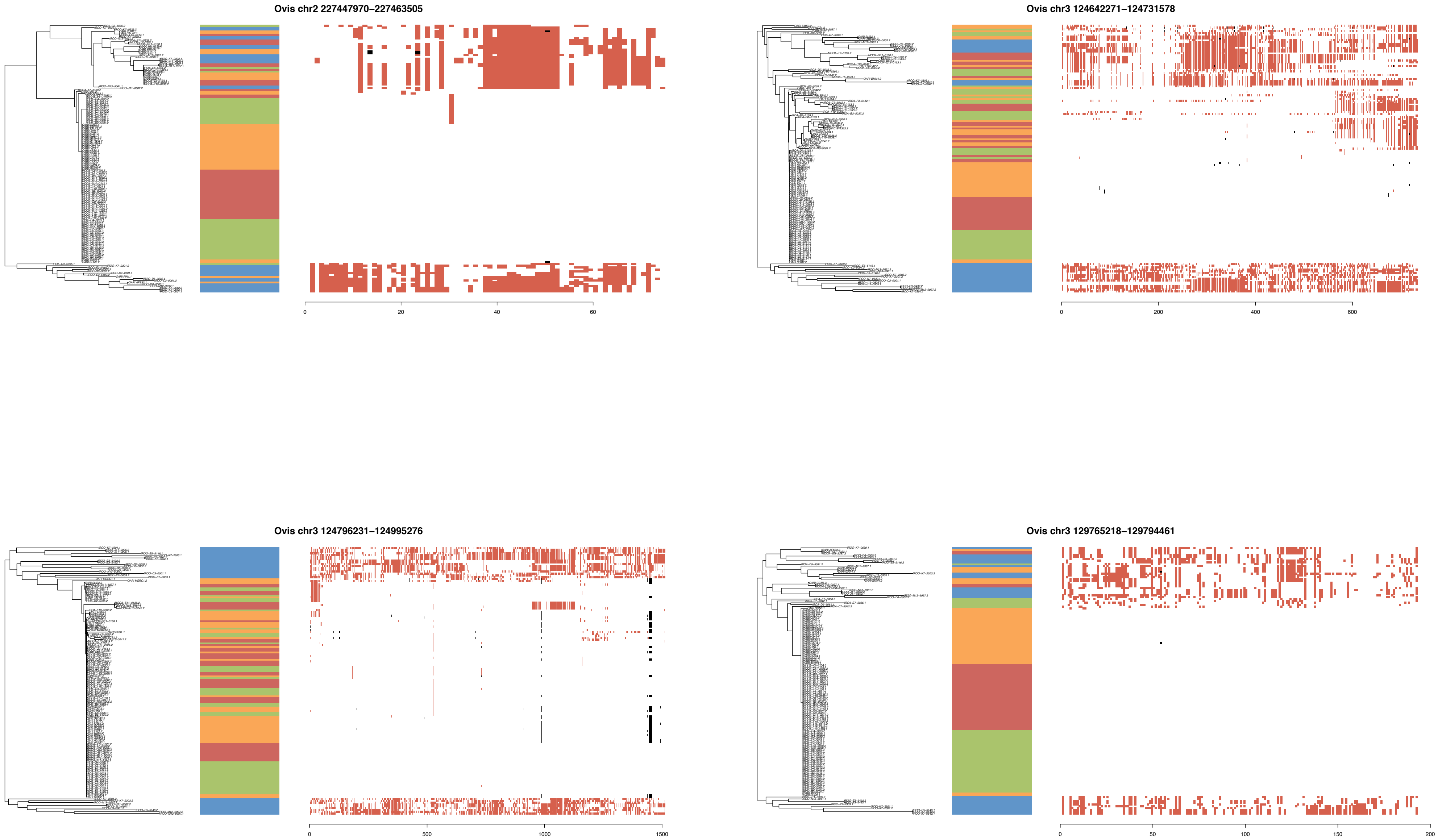

Supplementary Fig. 5 continued

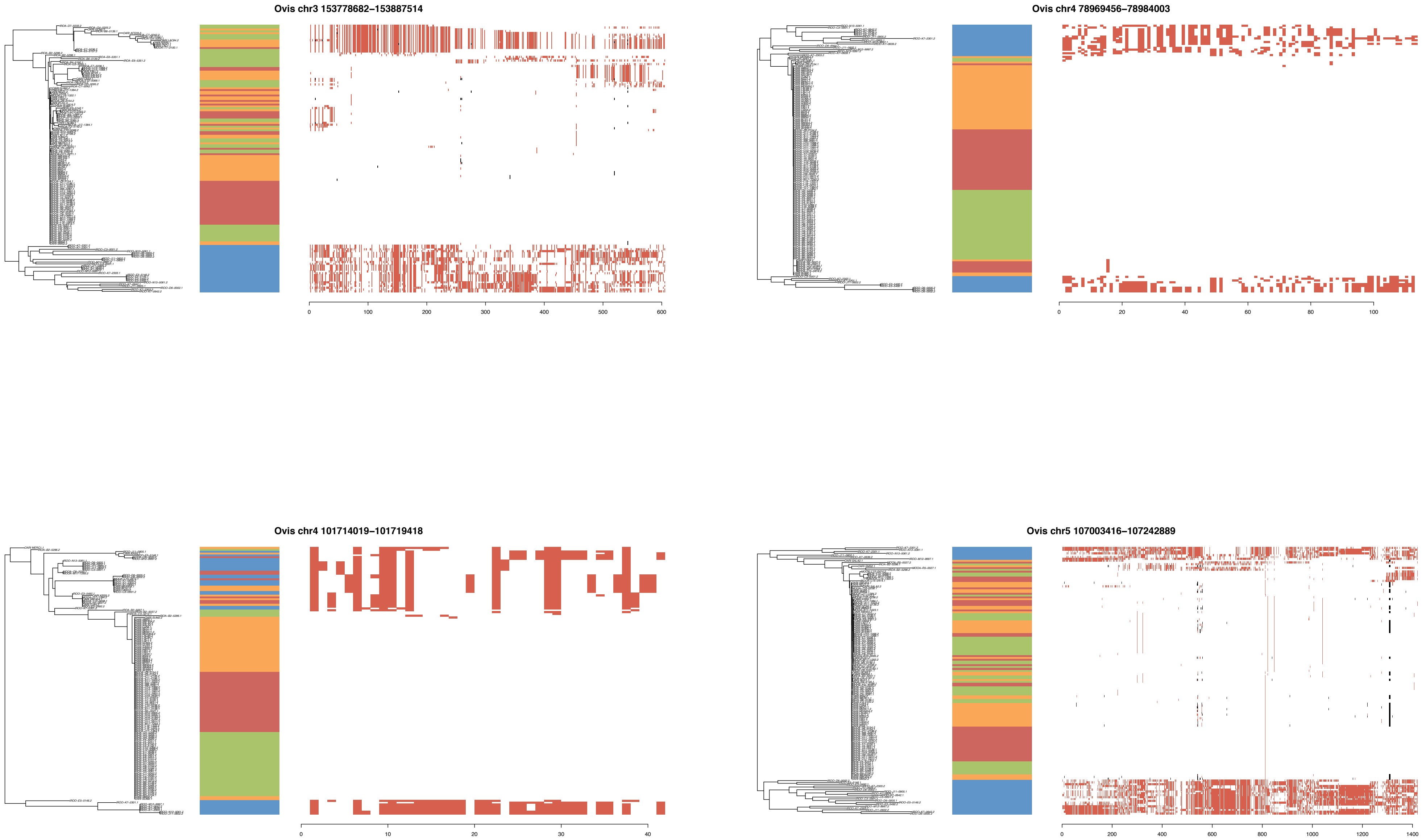

Supplementary Fig. 5 continued

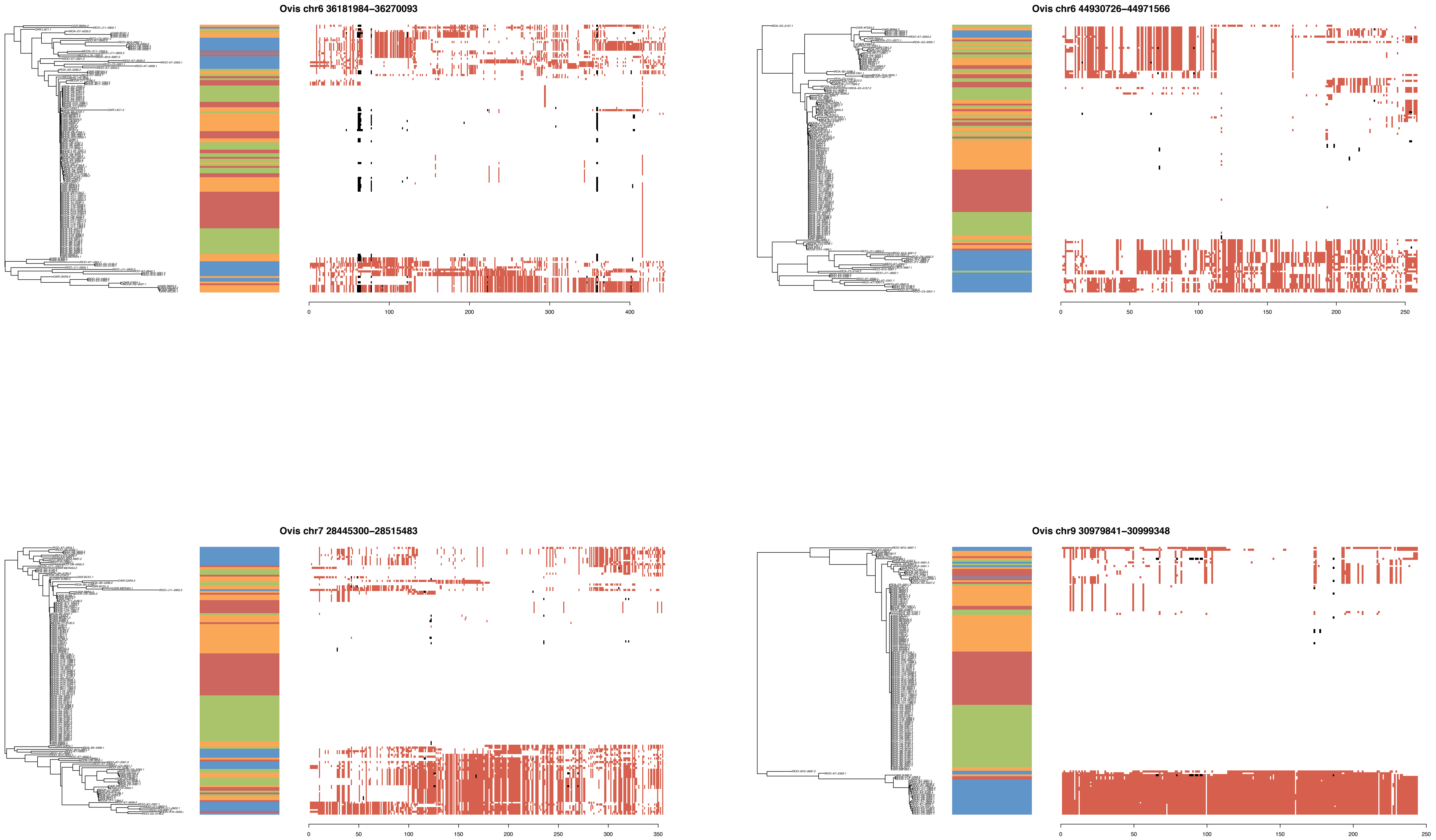

Supplementary Fig. 5 continued

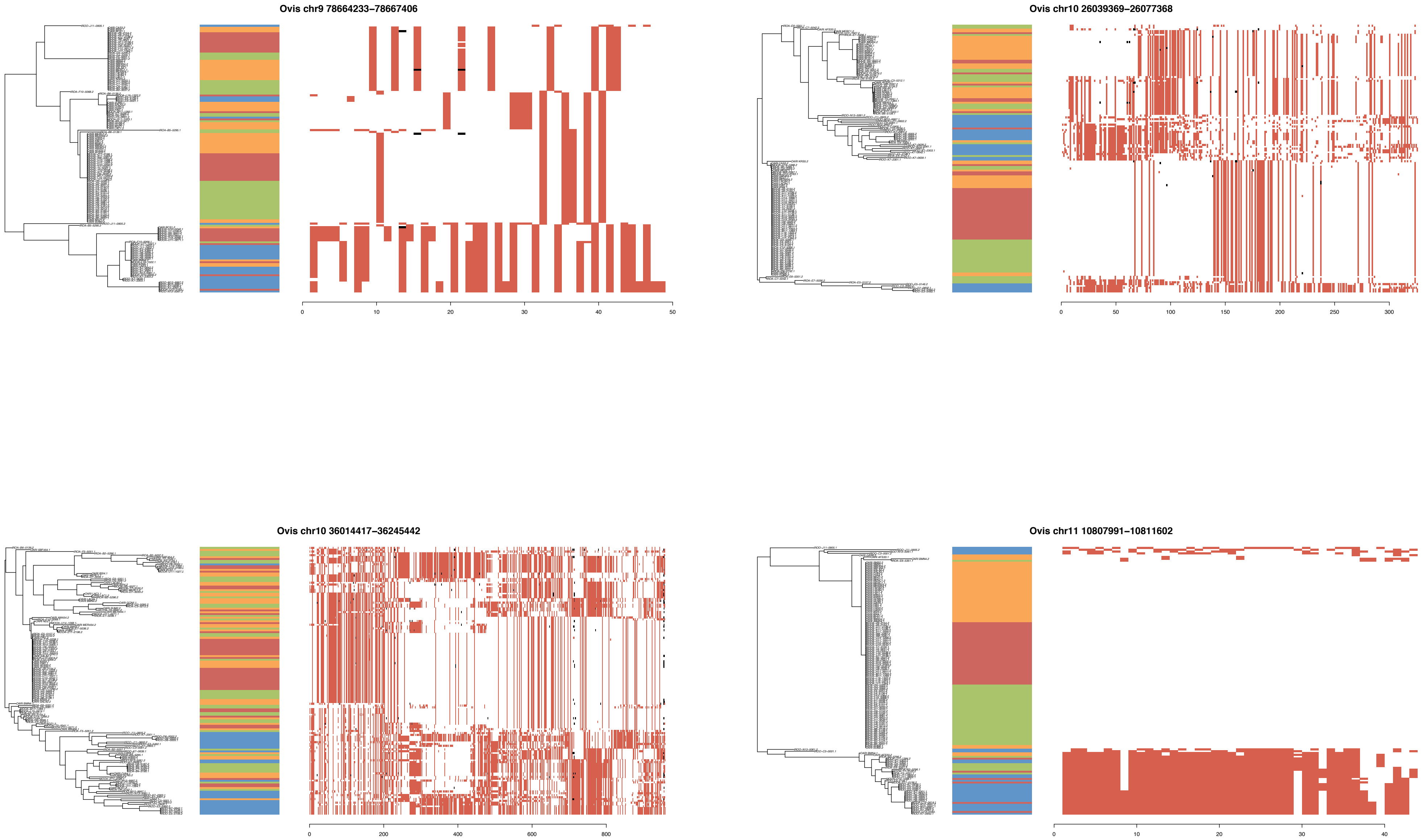

Supplementary Fig. 5 continued

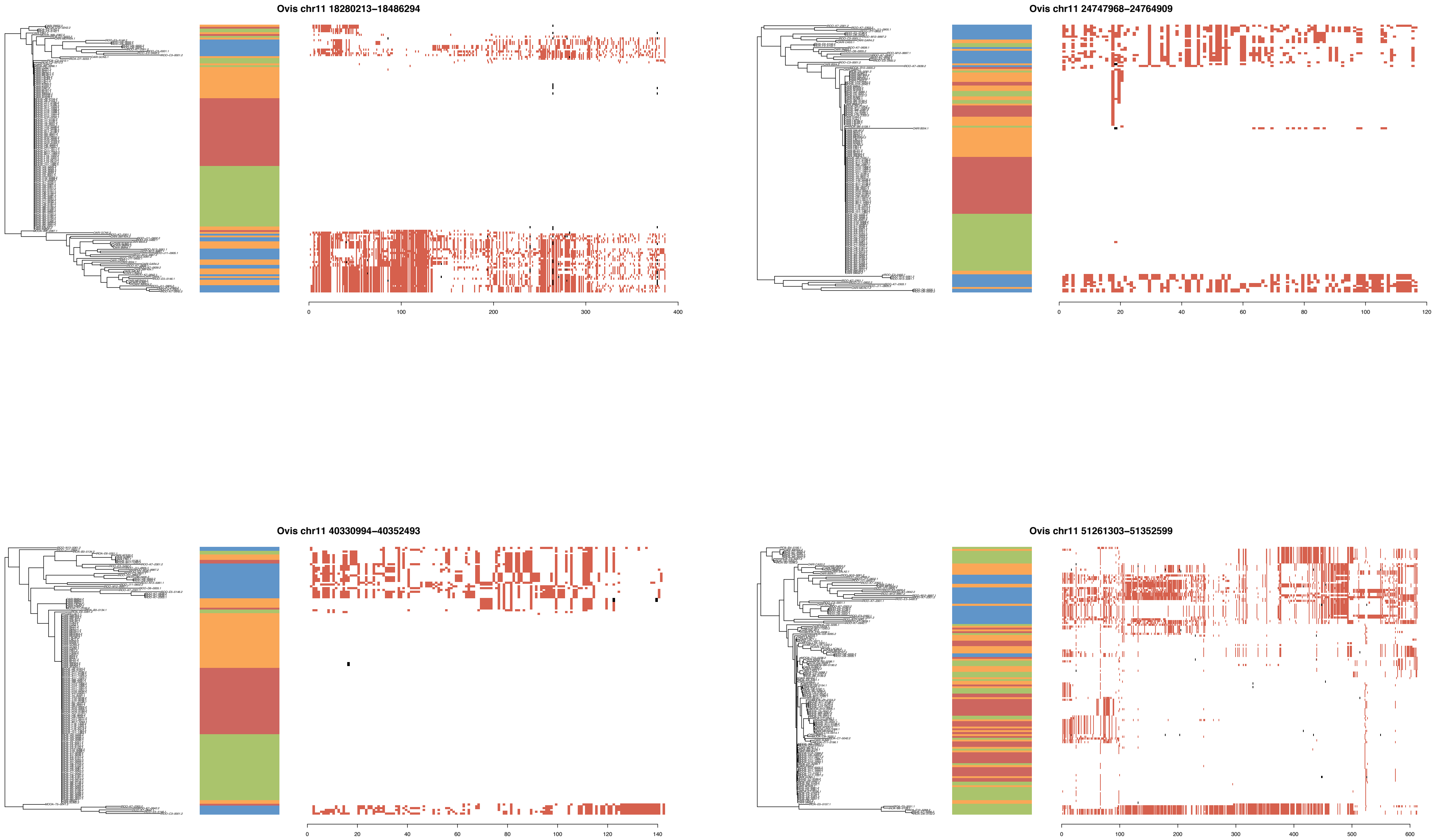

Supplementary Fig. 5 continued

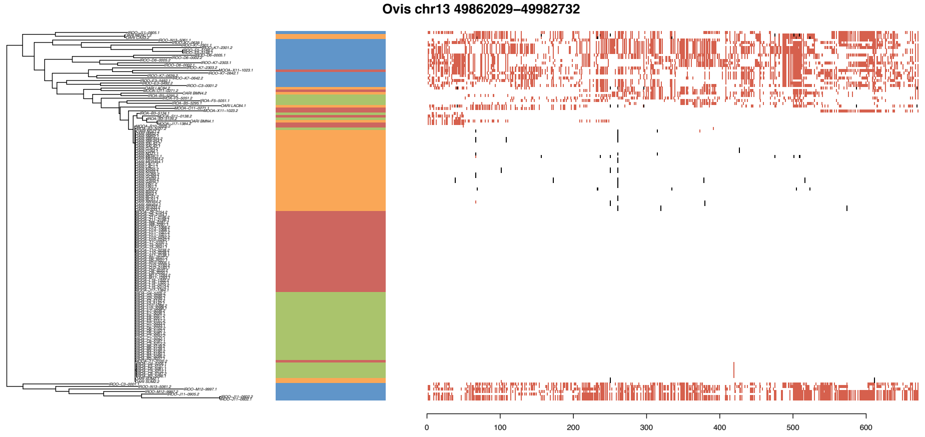

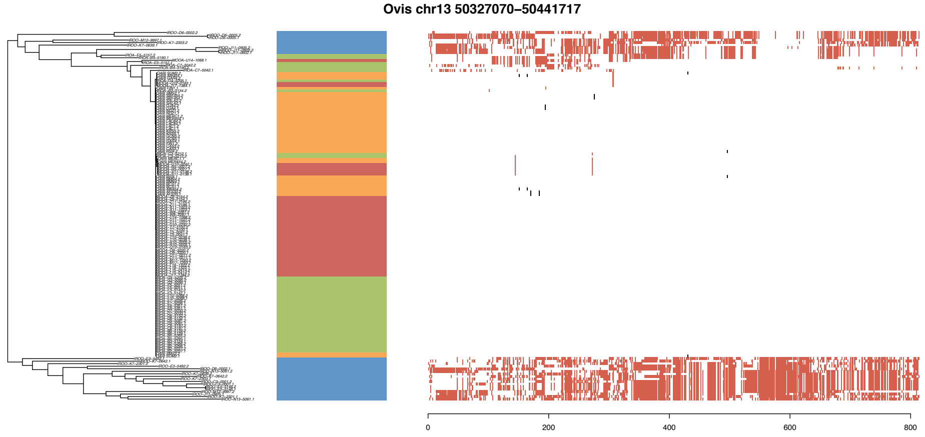

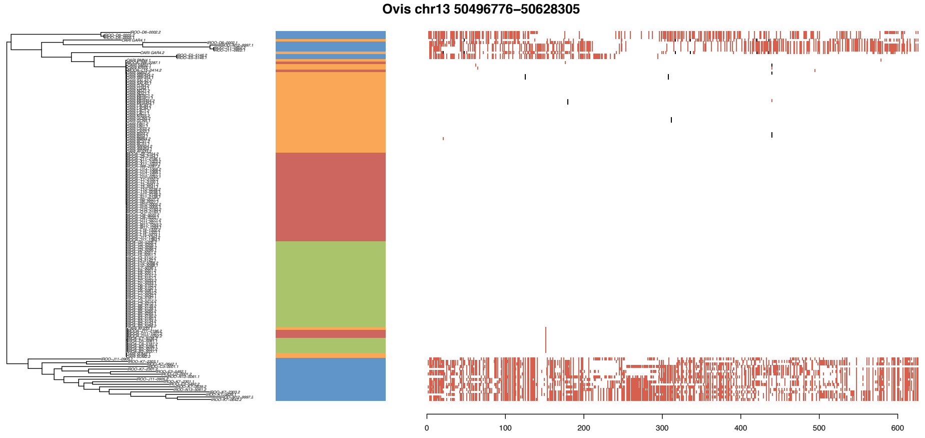

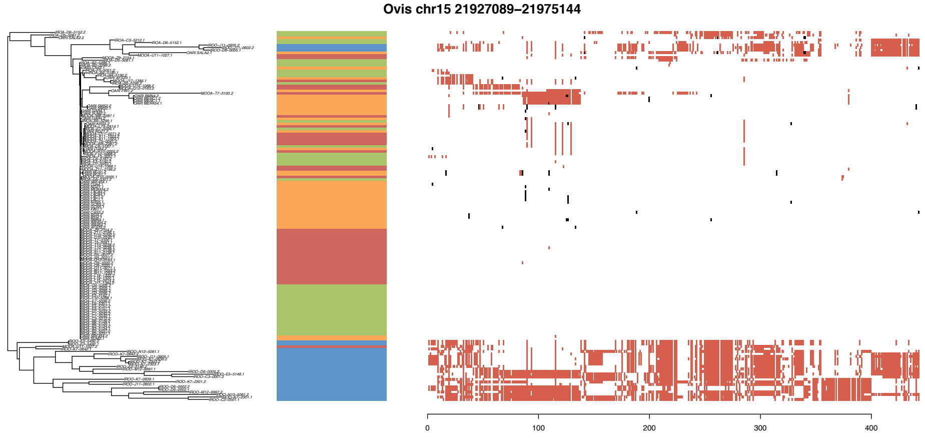

Supplementary Fig. 5 continued

Ovis chr15 47412823–47666895

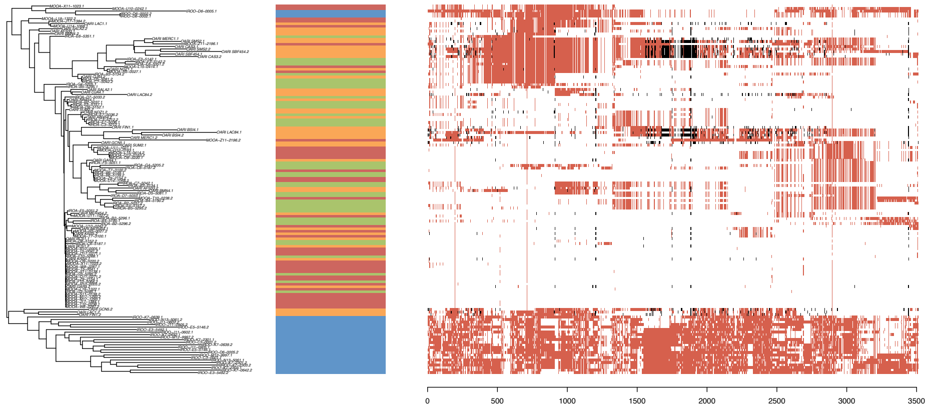

Ovis chr16 70895866–70976290

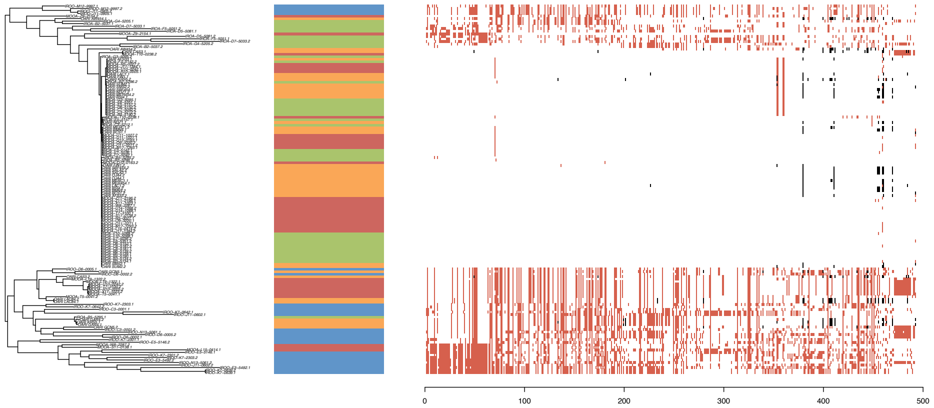

Ovis chr17 33093537–33109333

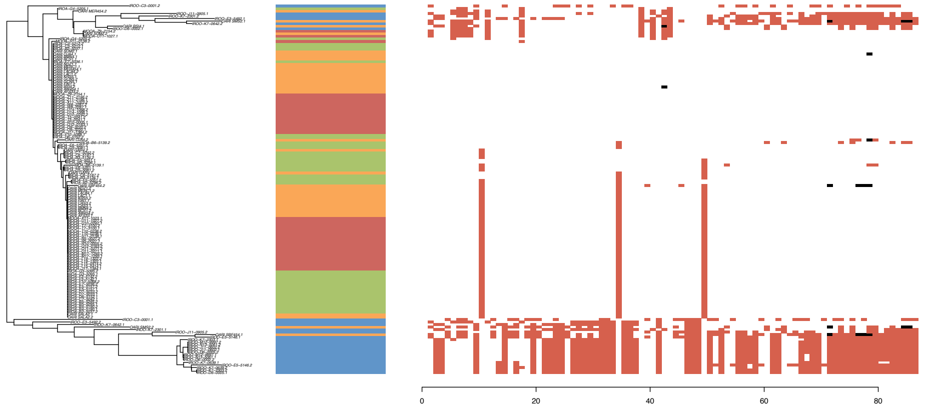

Ovis chr17 52416650–52440779

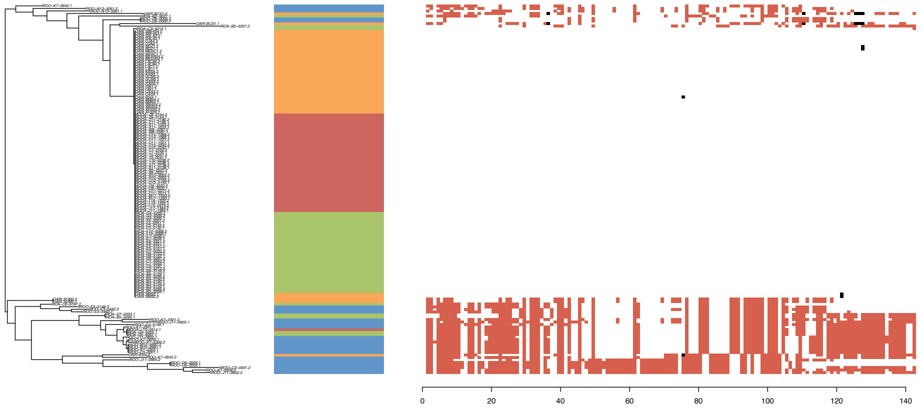

Supplementary Fig. 5 continued

Ovis chr17 59463489–59496869

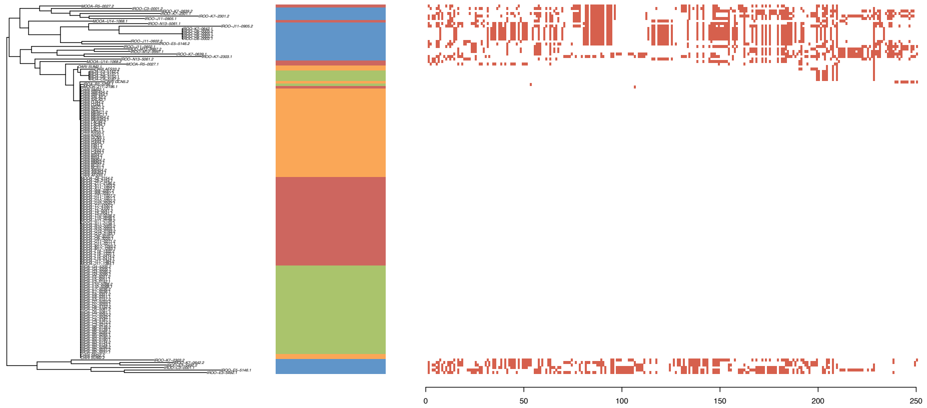

Ovis chr18 32242212–32391182

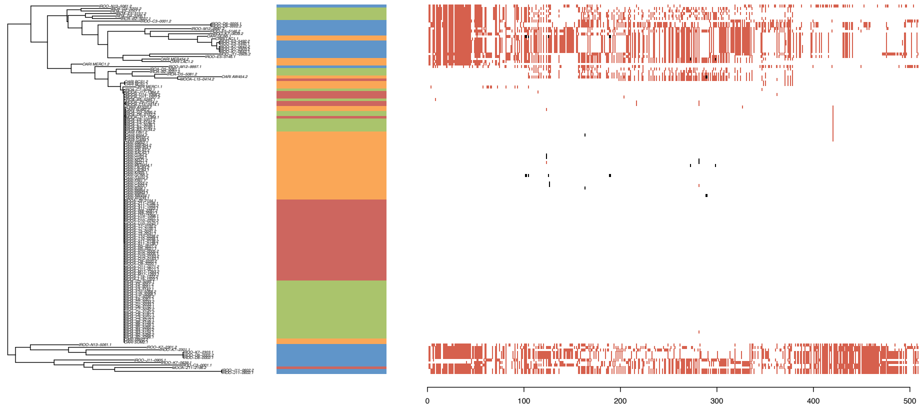

Ovis chr18 45533677–45554652

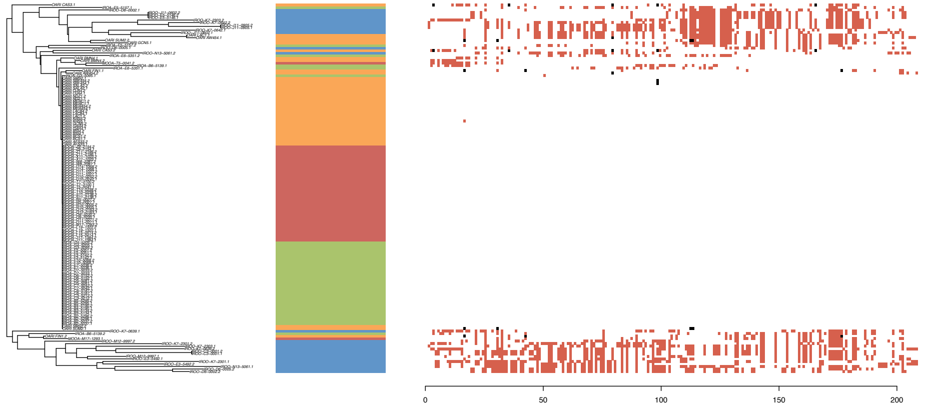

Ovis chr20 15048734–15202179

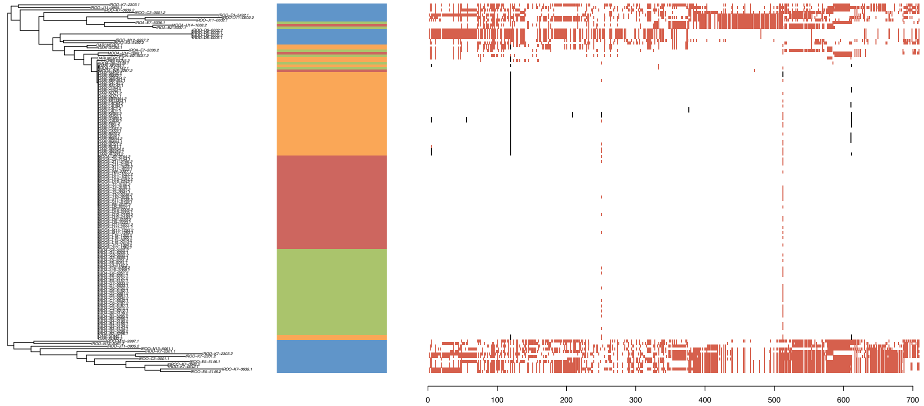

Supplementary Fig. 5 continued

Ovis chr20 18330310–18449122

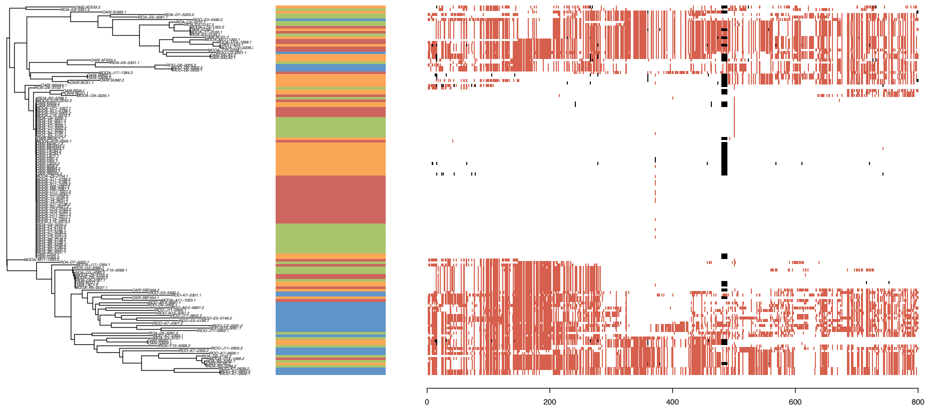

Ovis chr20 50132457–50153376

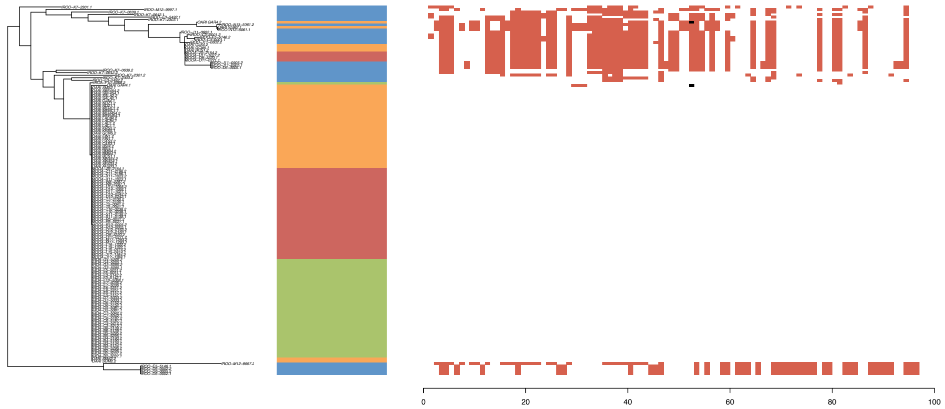

Ovis chr20 50909969–51063580

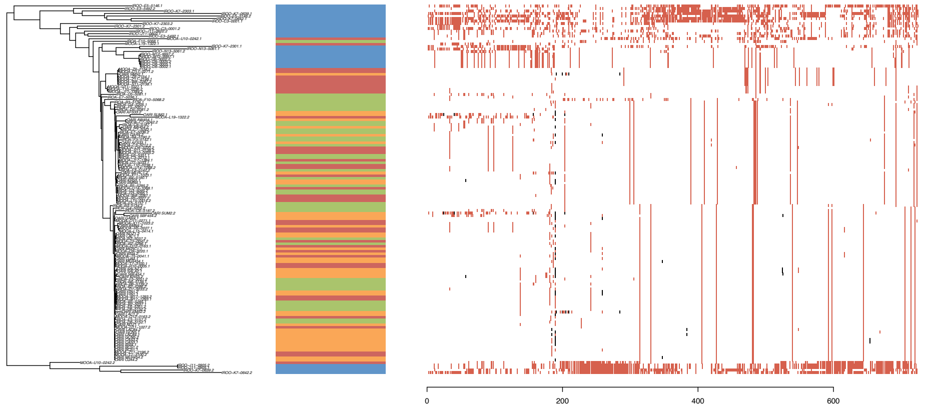

Ovis chr21 49681861–49870927

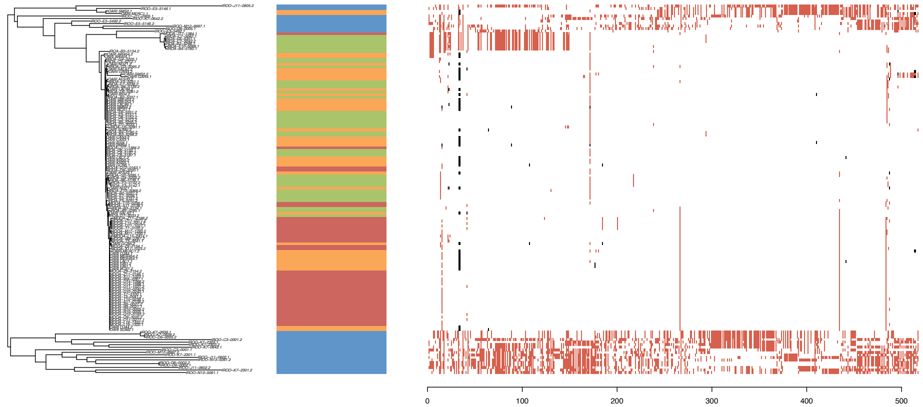

Supplementary Fig. 5 continued

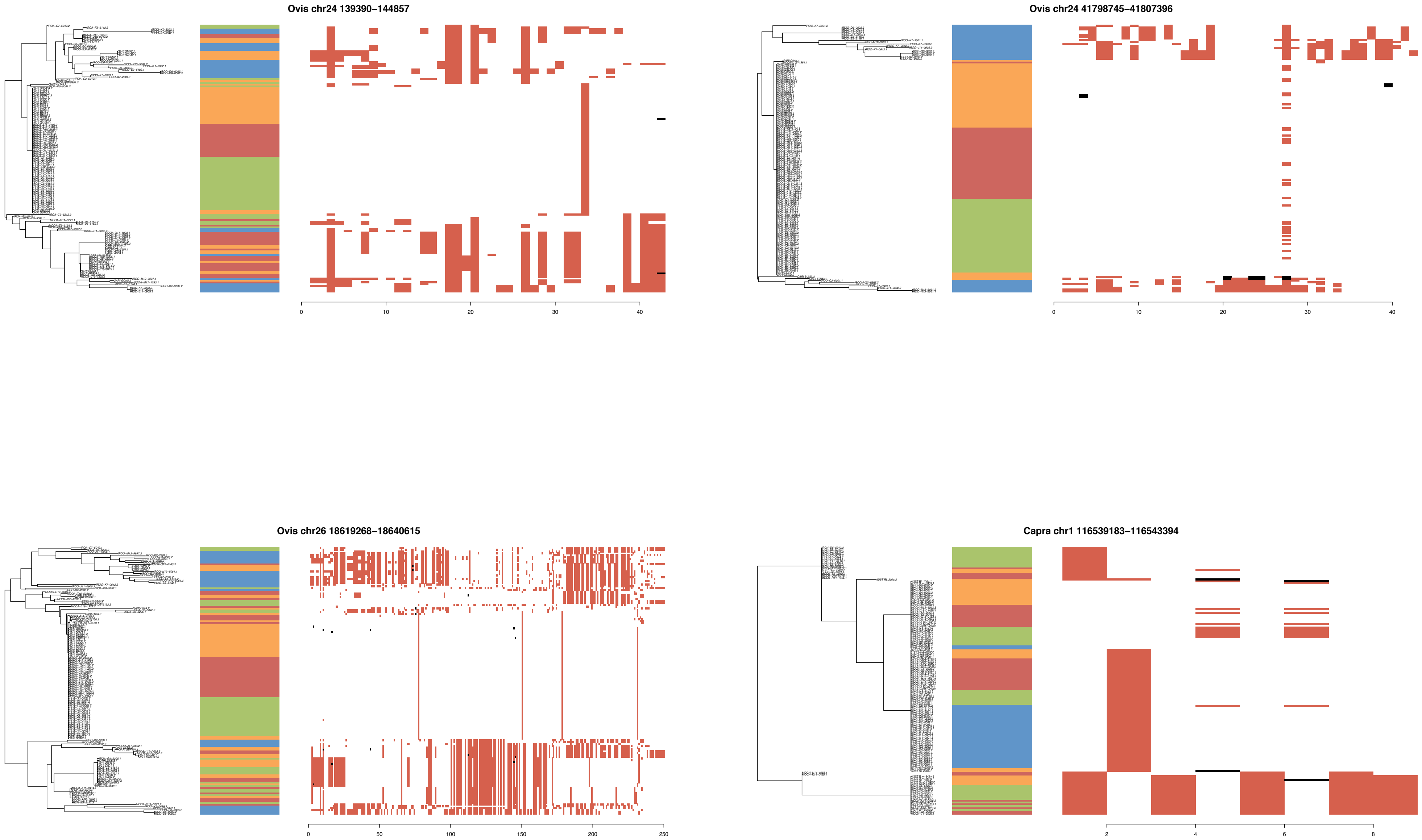

Supplementary Fig. 5 continued

Capra chr1 118998939–119026674

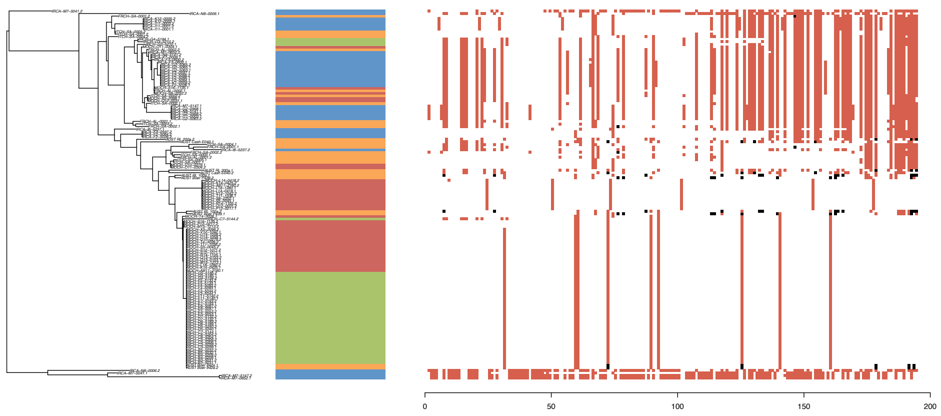

Capra chr2 113837943–113955464

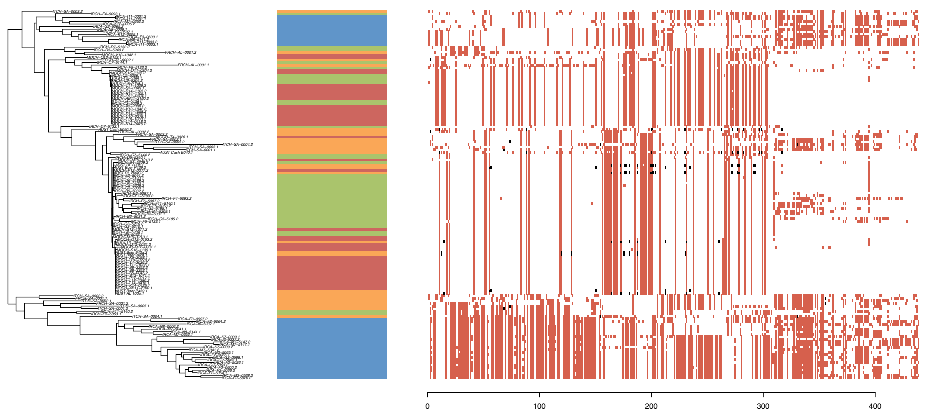

Capra chr3 8424129–8490639

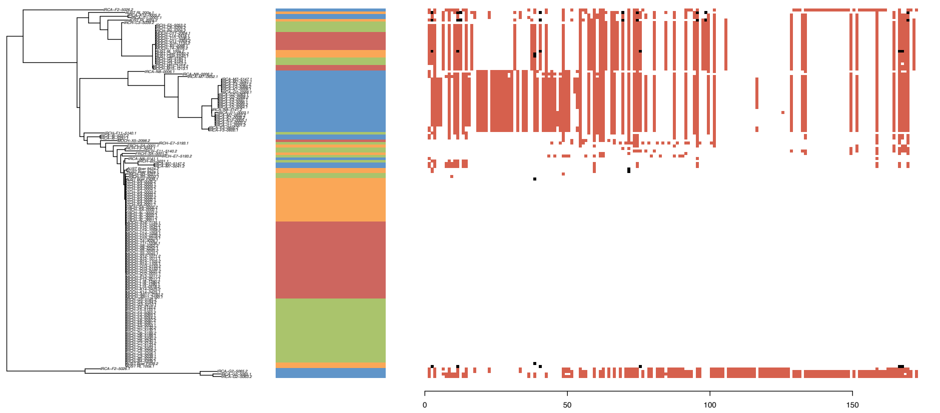

Capra chr3 11781186–12781186

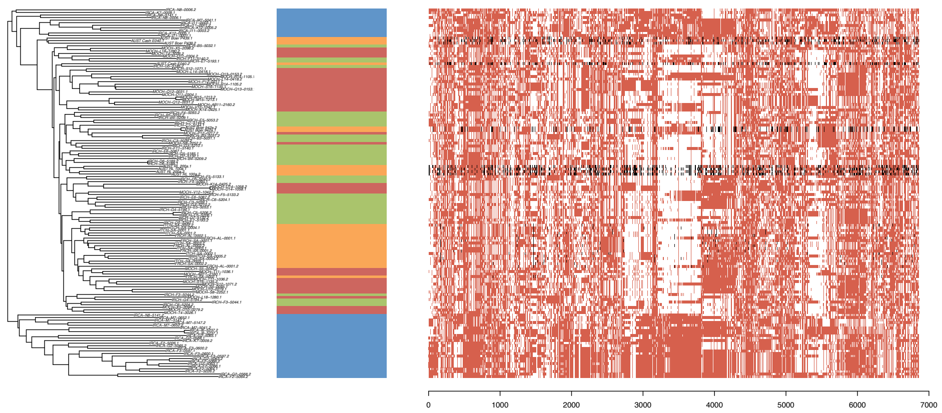

Supplementary Fig. 5 continued

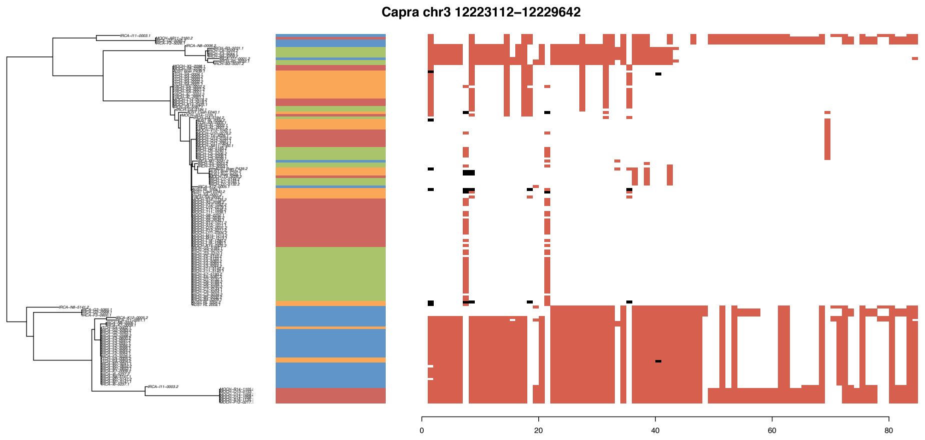

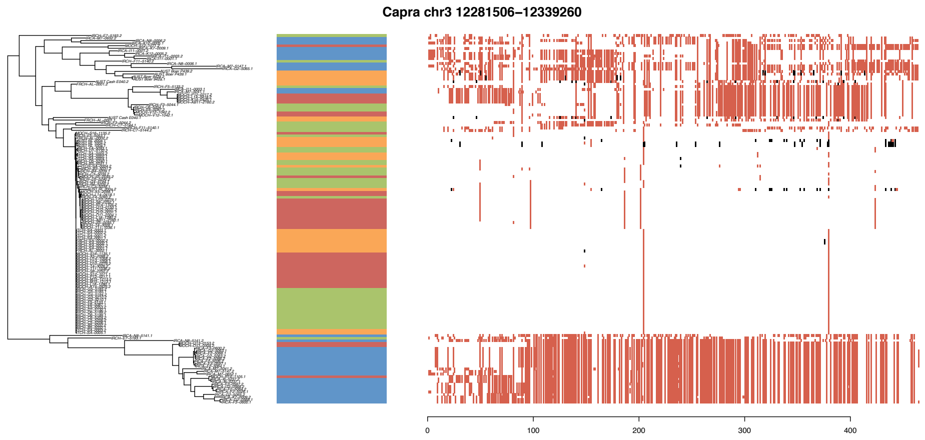

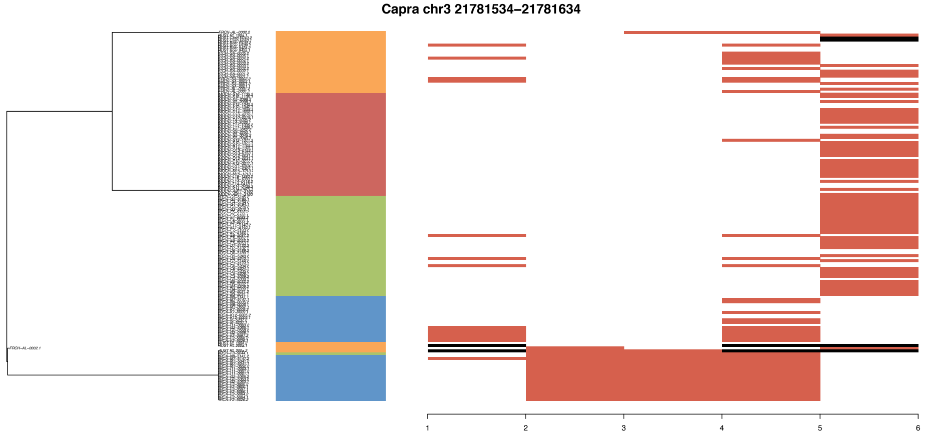

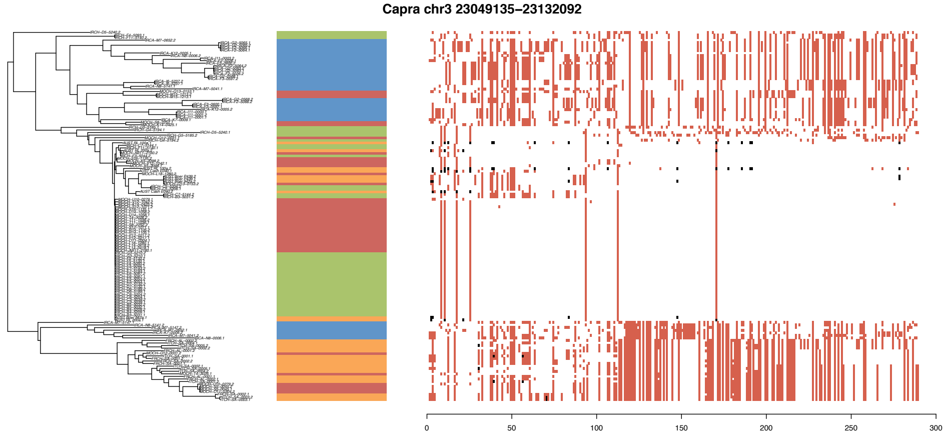

Supplementary Fig. 5 continued

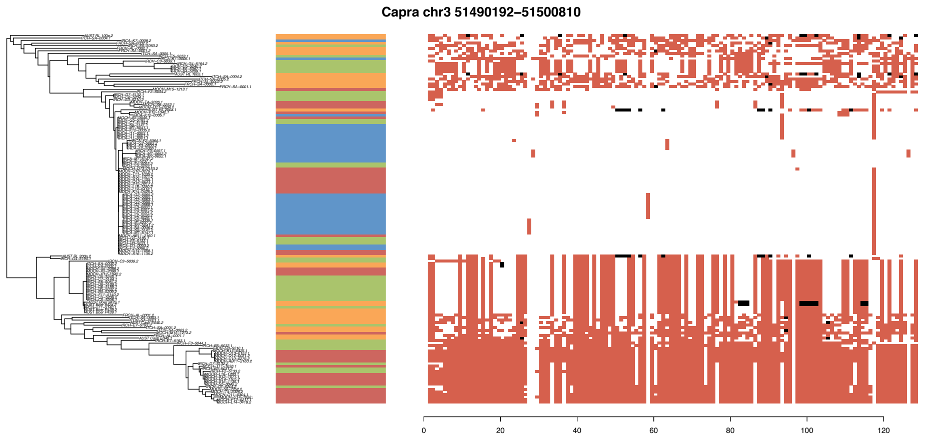

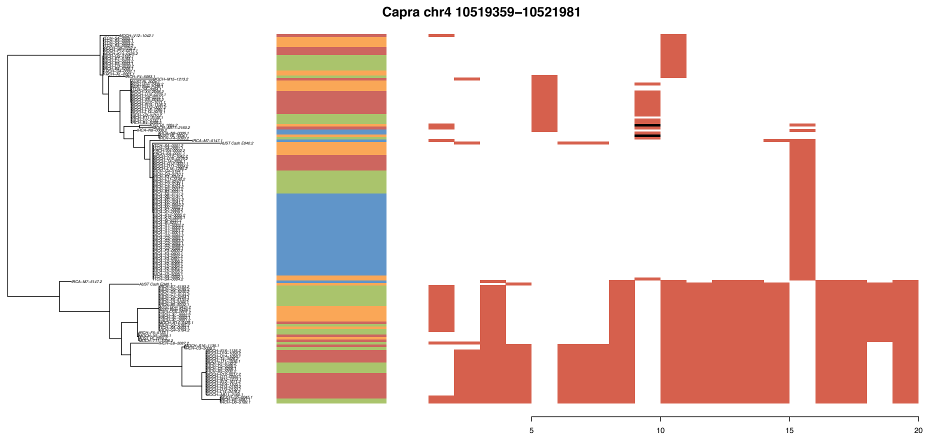

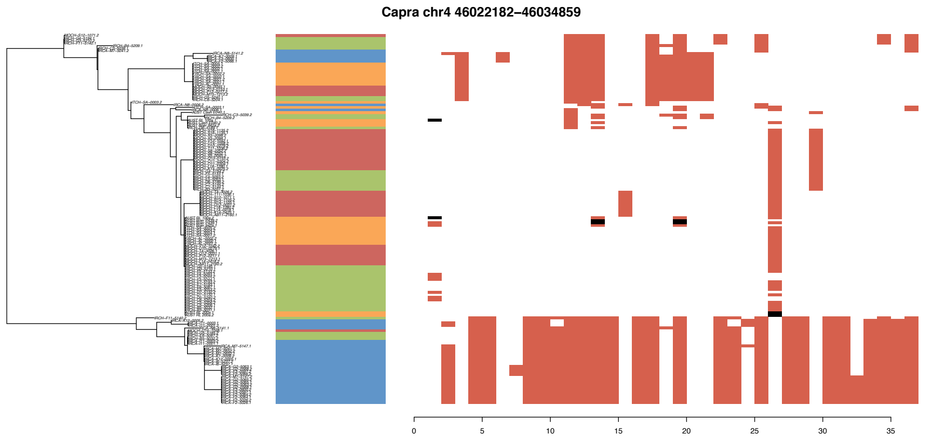

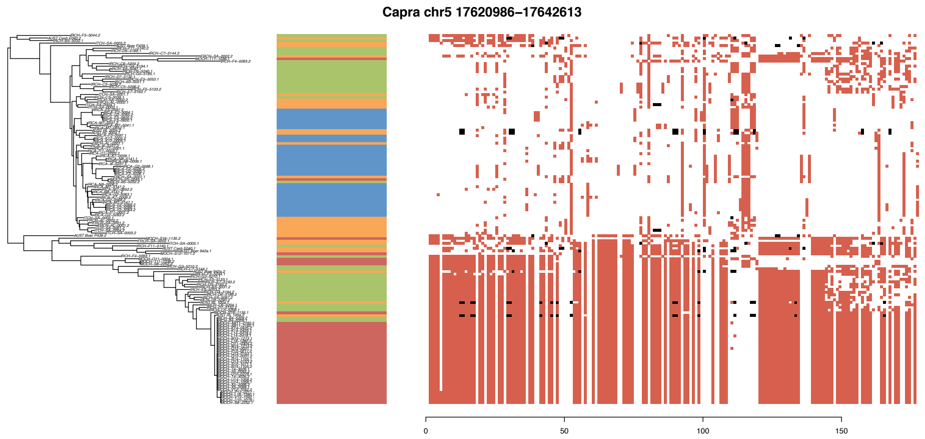

Supplementary Fig. 5 continued

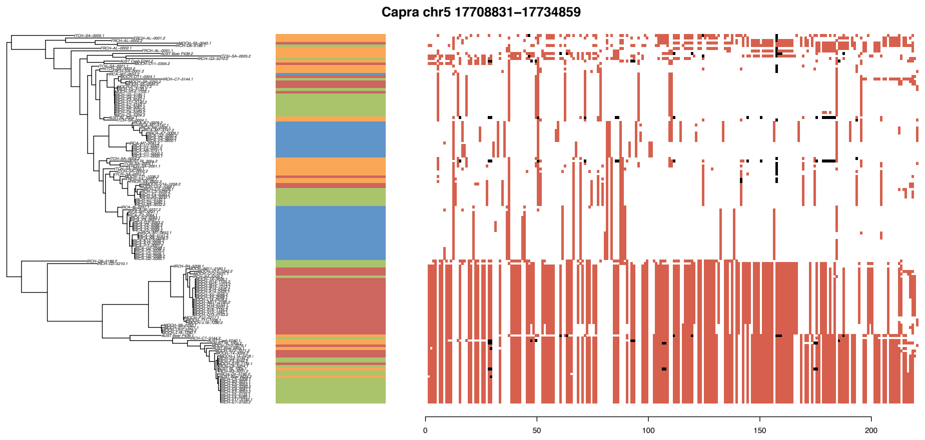

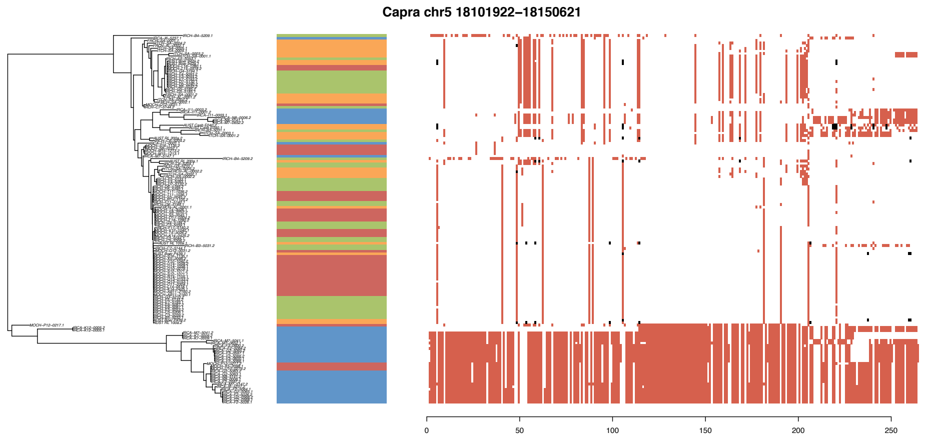

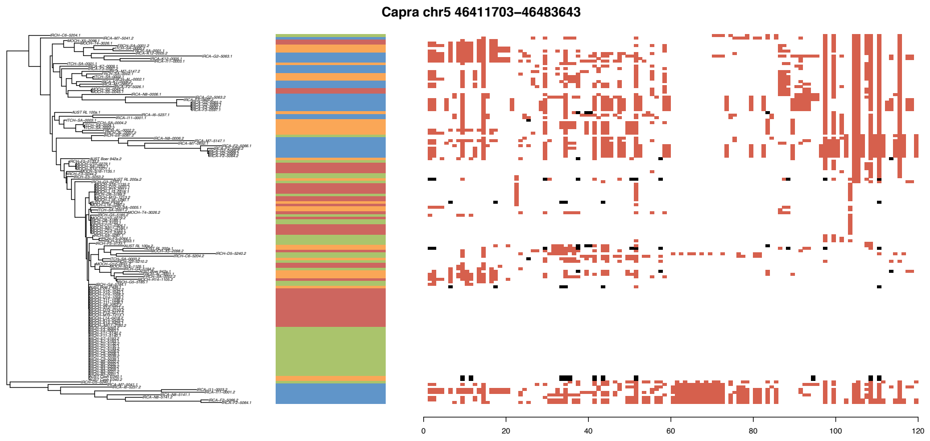

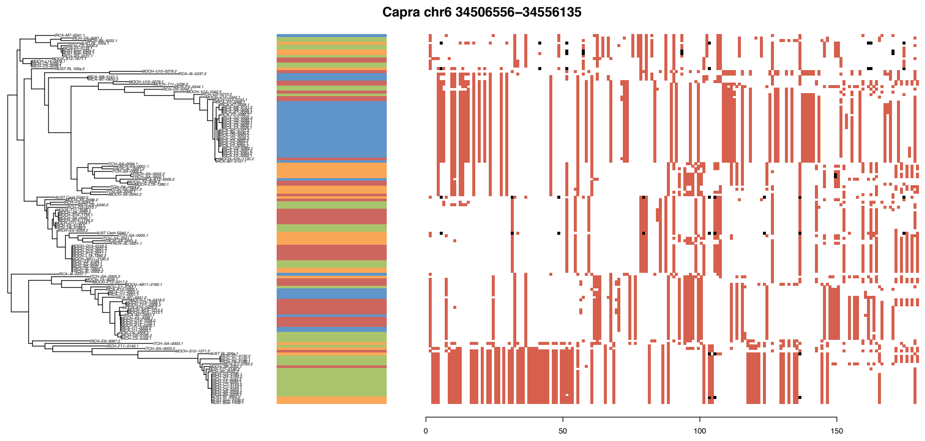

Supplementary Fig. 5 continued

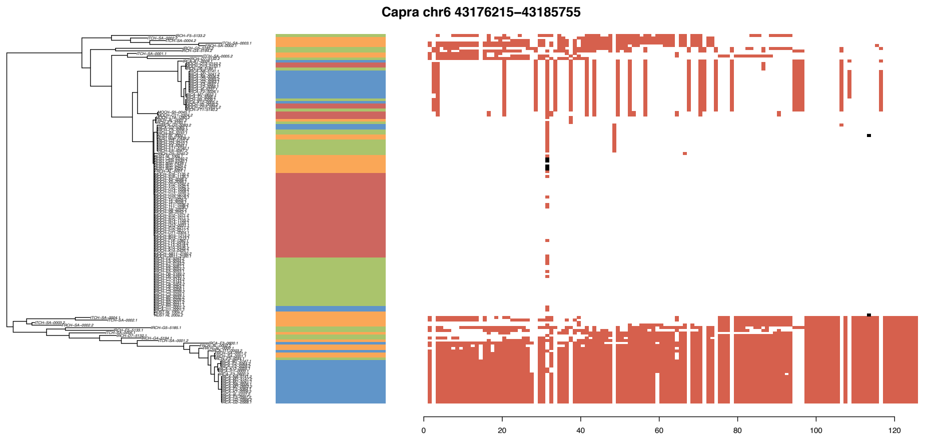

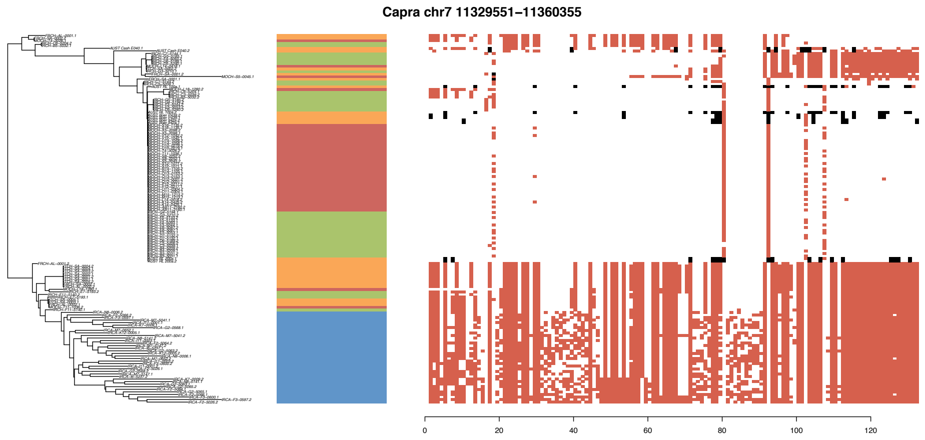

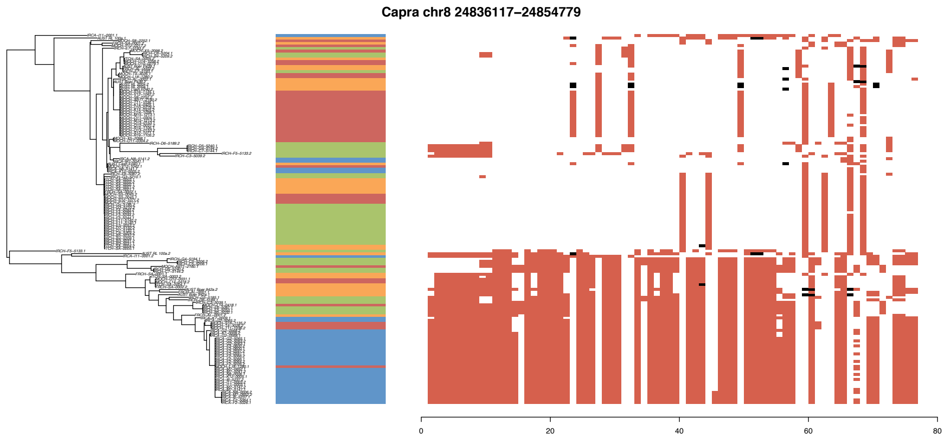

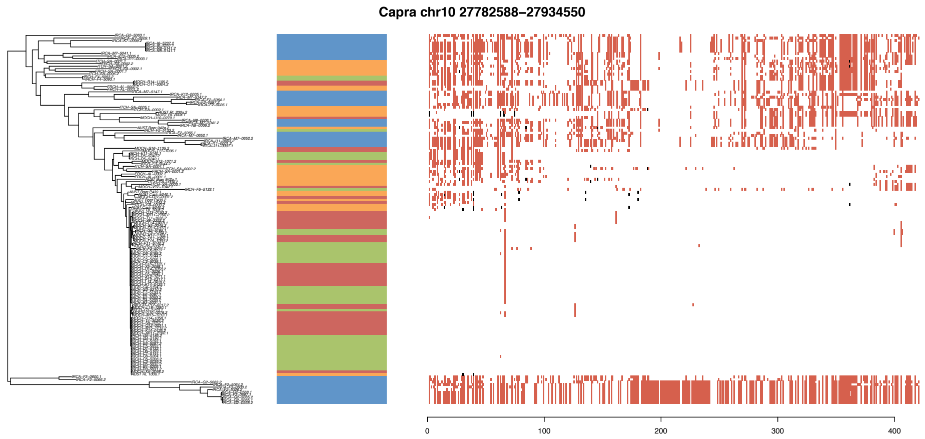

Supplementary Fig. 5 continued

Capra chr11 67276335–67294129

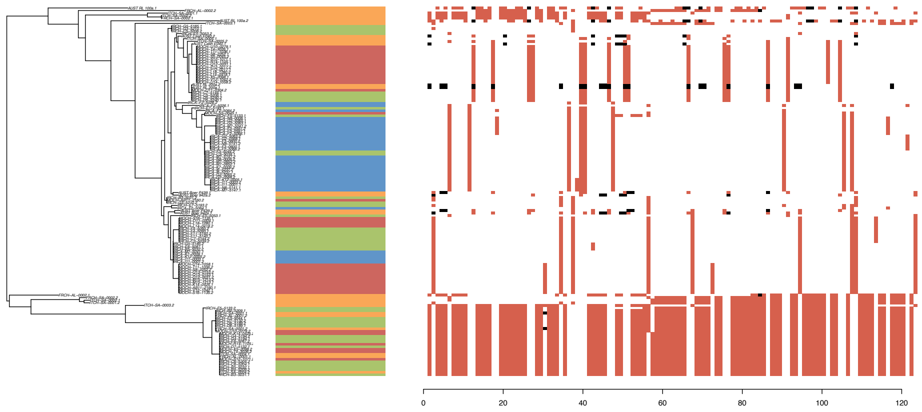

Capra chr11 93456660–93545455

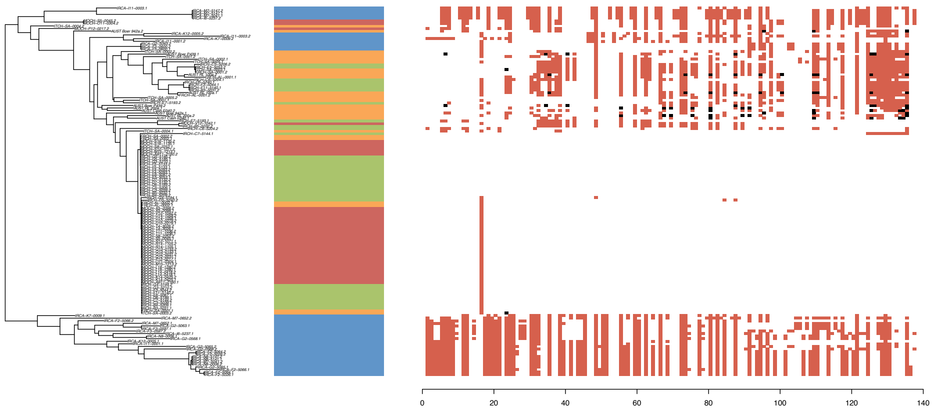

Capra chr12 23572775–23576205

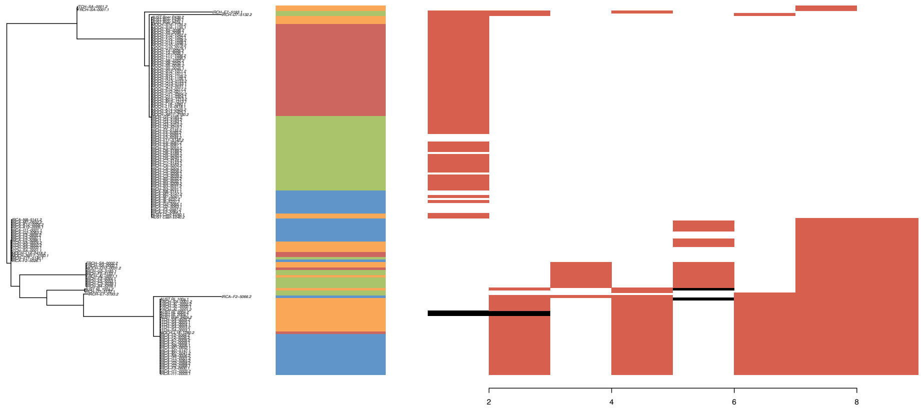

Capra chr12 33607541–33842316

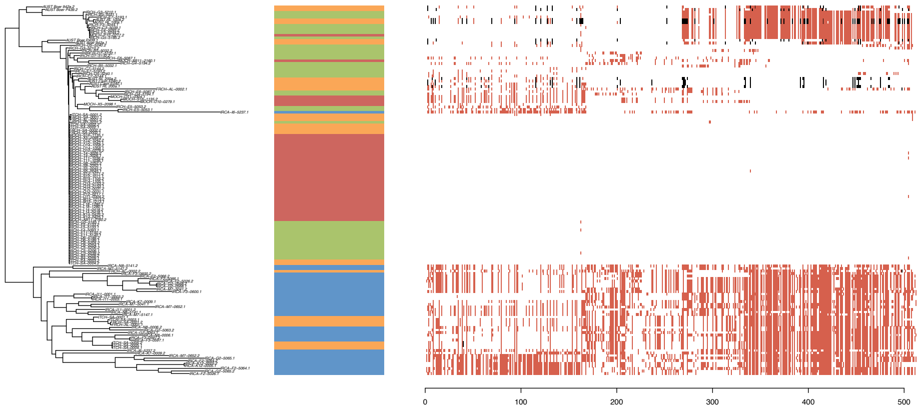

Supplementary Fig. 5 continued

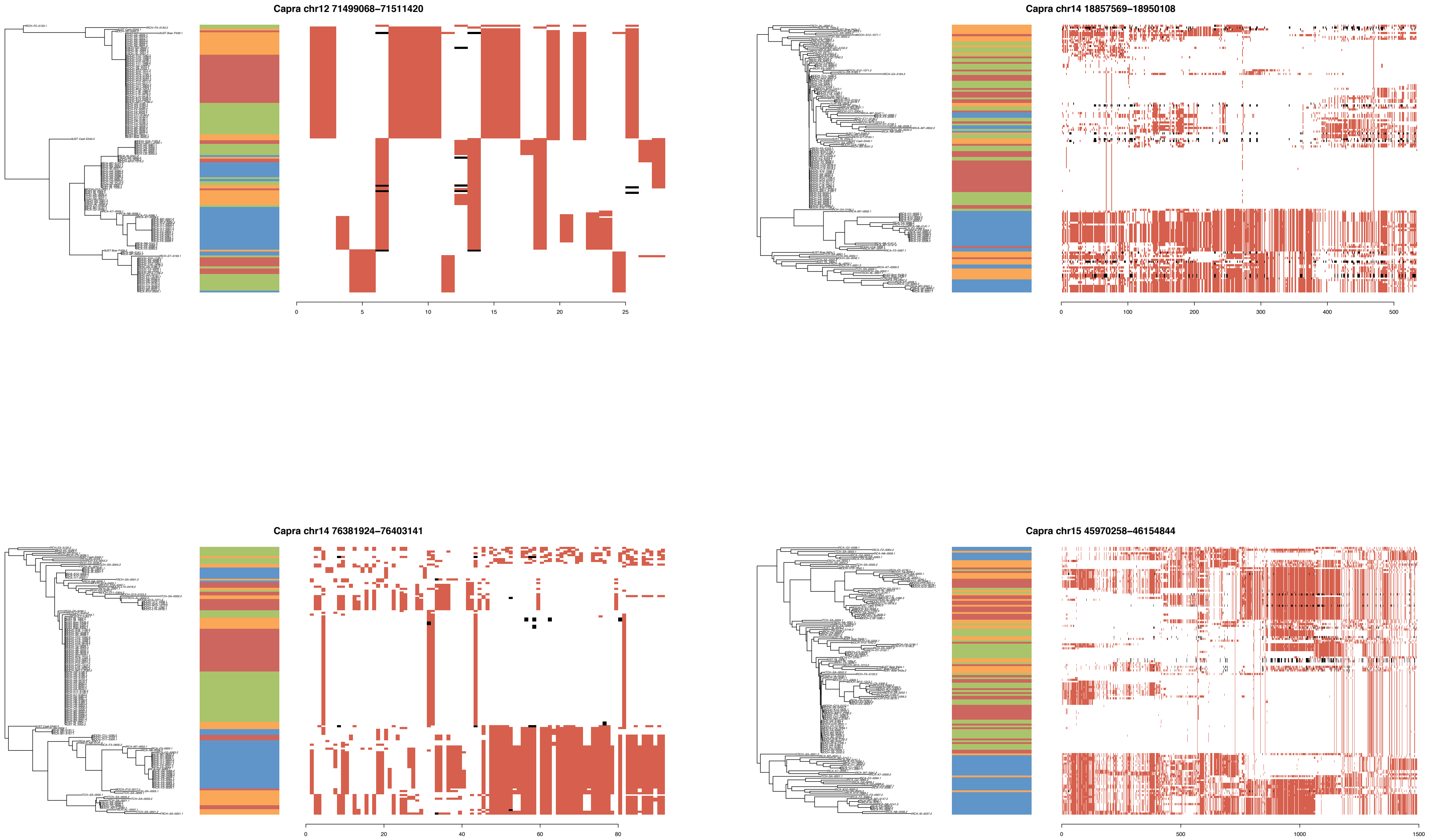

Supplementary Fig. 5 continued

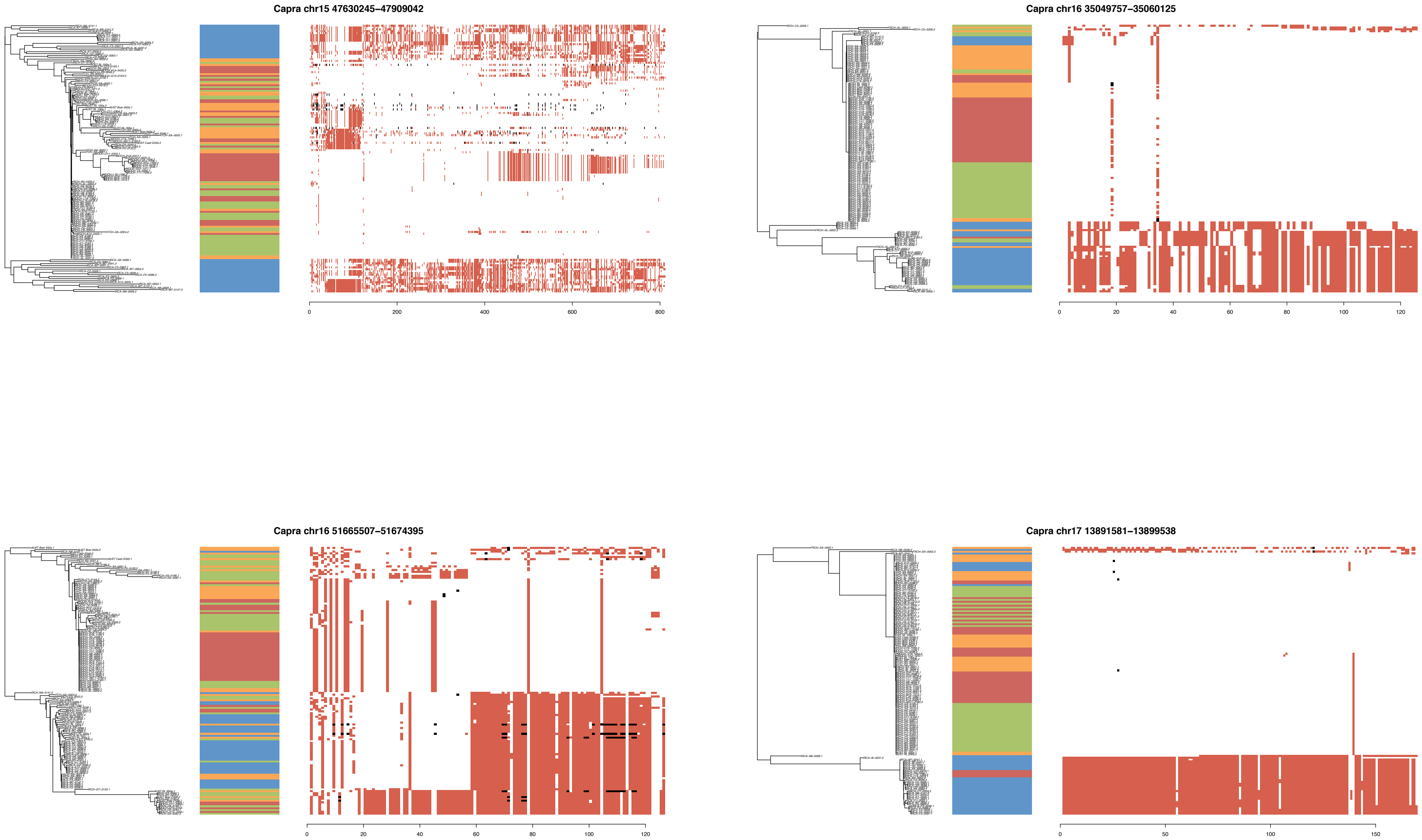

Supplementary Fig. 5 continued

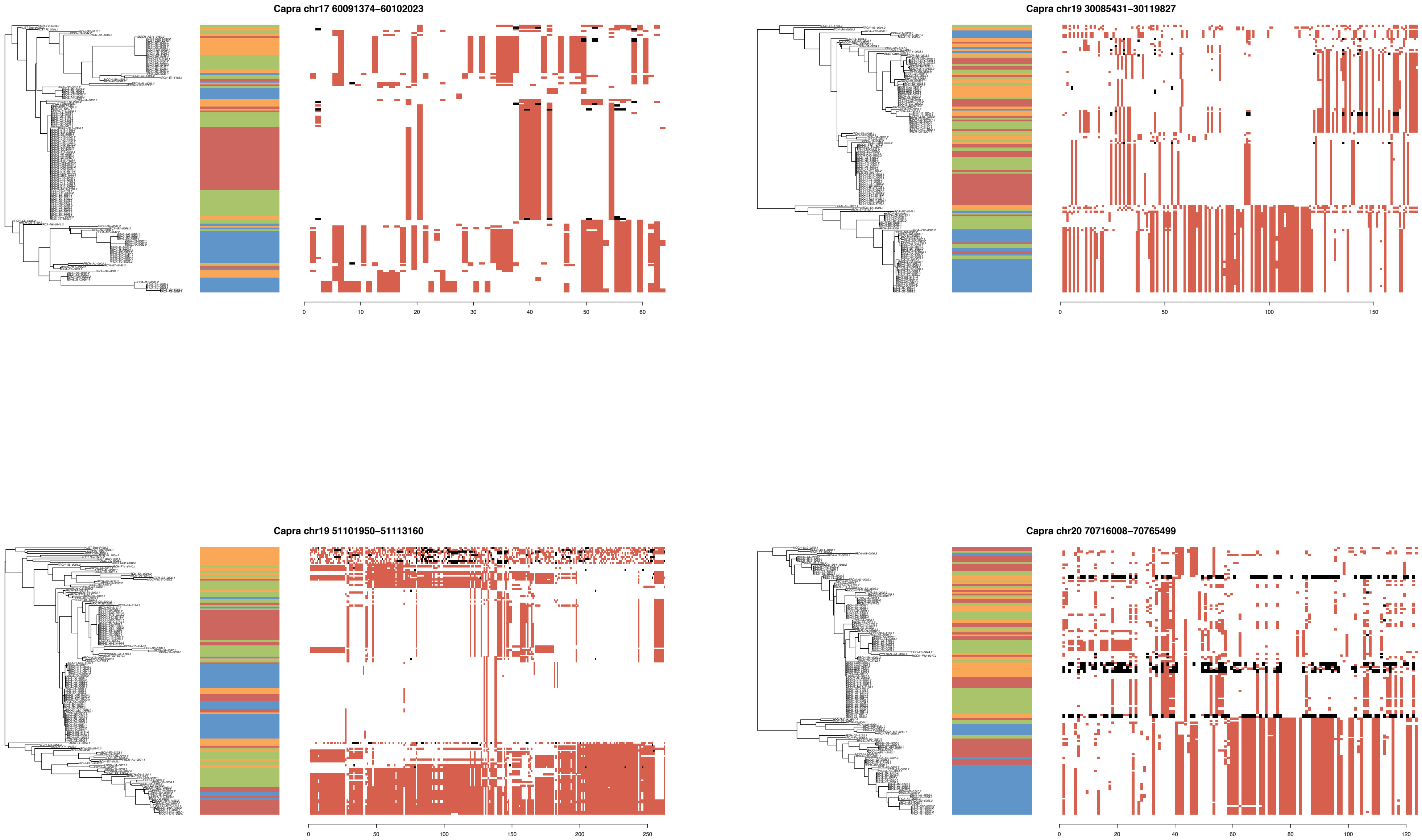

Supplementary Fig. 5 continued

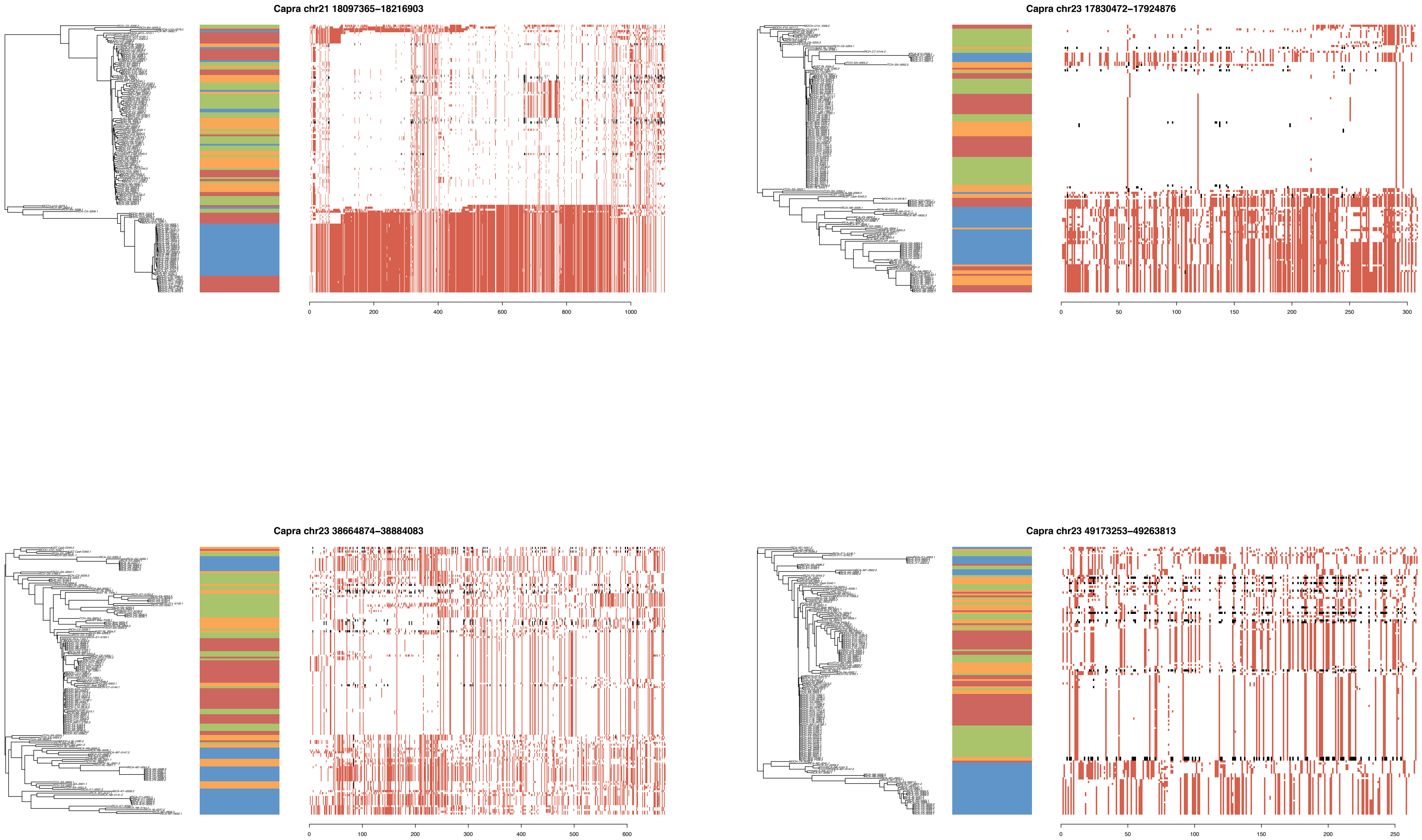

Supplementary Fig. 5 continued

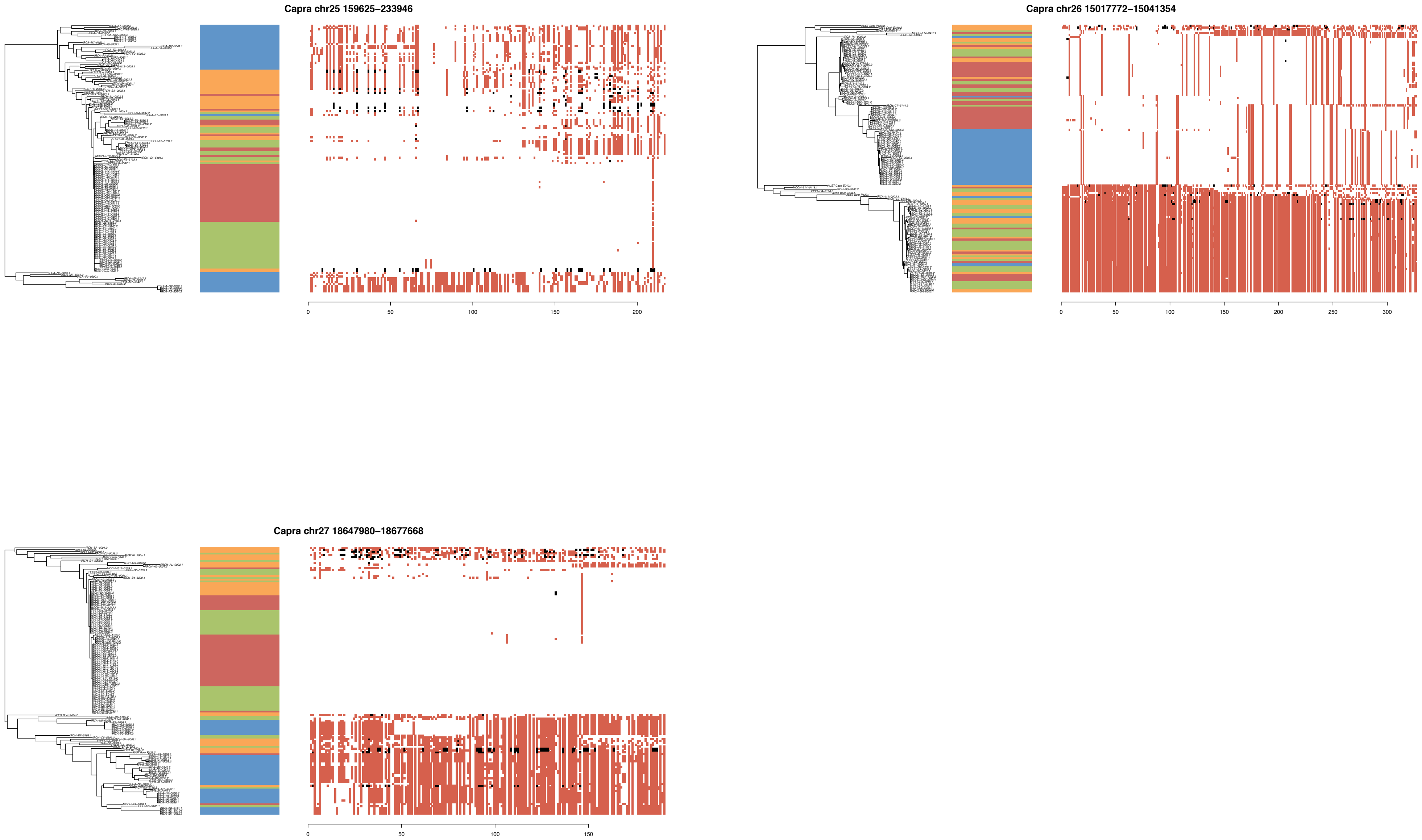

Supplementary Fig. 5: Midpoint rooted Neighbour-Joining trees based on the % of identity between sequences for regions under selection in Ovis and Capra.

The 2n haplotypes are represented as leaf/row in the panels. The colour bars (center panel) relates each haplotype to its group (blue: wild, green: Iranian domestics, red: Moroccan domestics, orange: world panel). The right panel depict all SNPs in the considered haplotype (brown and blue squares represent minor alleles. Black squares represent missing data).

• **Supplementary Figure 6**

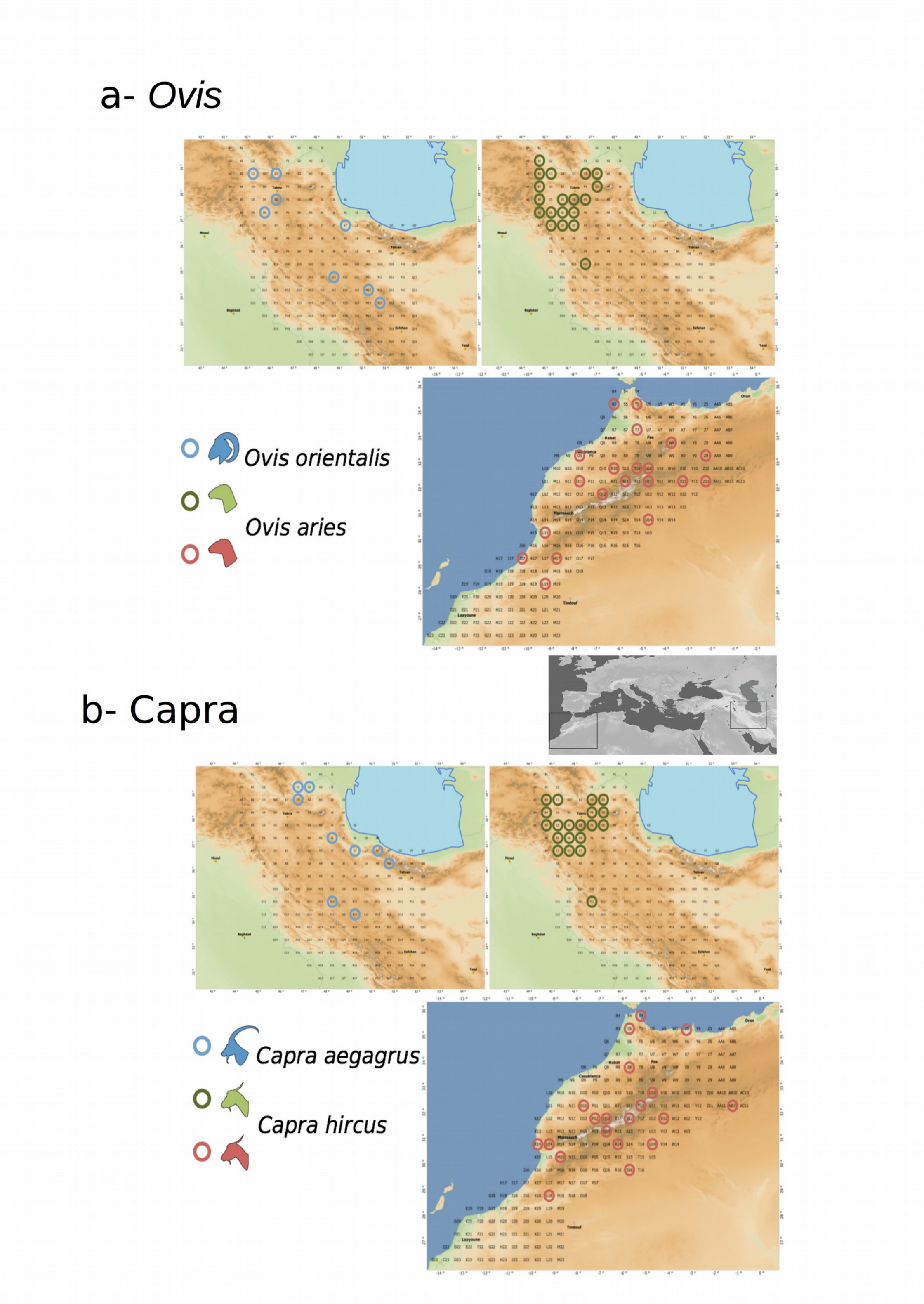

Supplementary Fig. 6: Sampling positions in Iran and Morocco for *Ovis* and *Capra*.

• Supplementary Figure 7

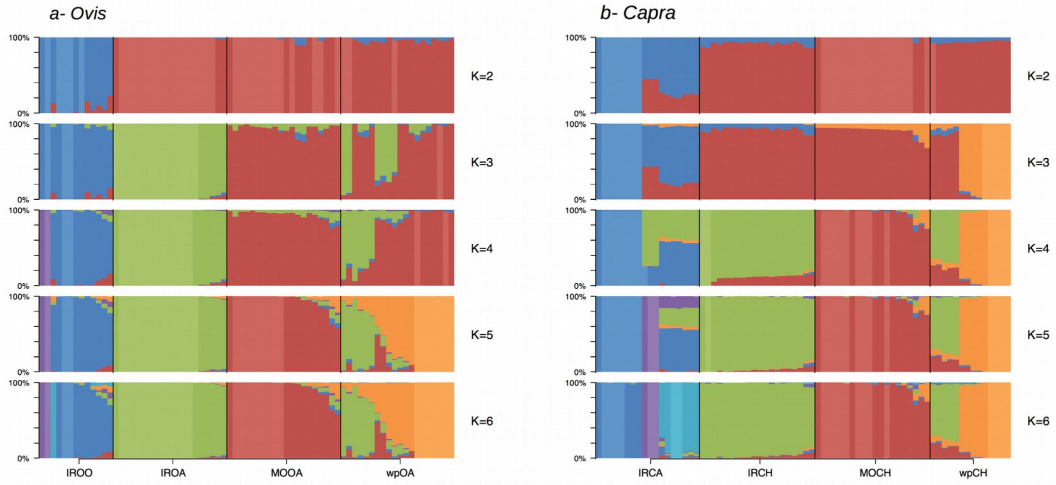

• Supplementary Fig. 7: Clustering by genetic ancestry using sNMF with increasing number of ancestral populations (K) for *Ovis* and *Capra*.

• **Supplementary Fig. 8**

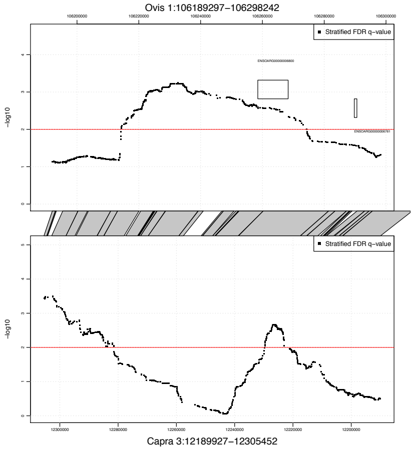

ENSOARG00000006800

Other (Uncharacterised)

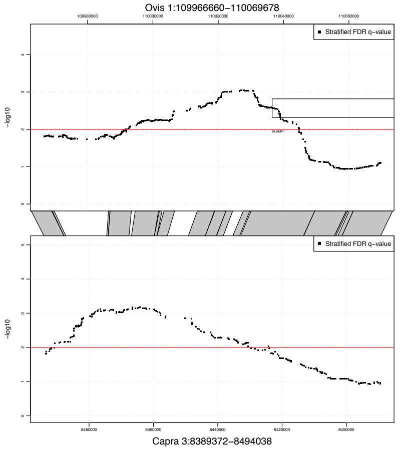

SLAMF1

Immunity

SLAMF1

Immunity

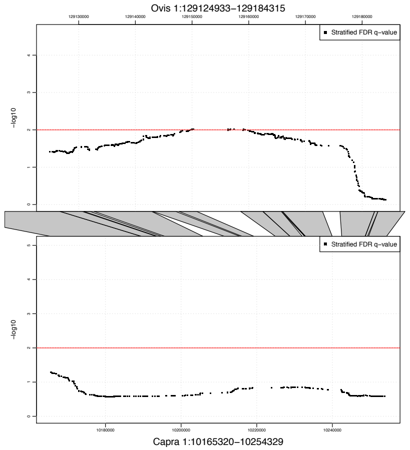

Intergenic

Unknown

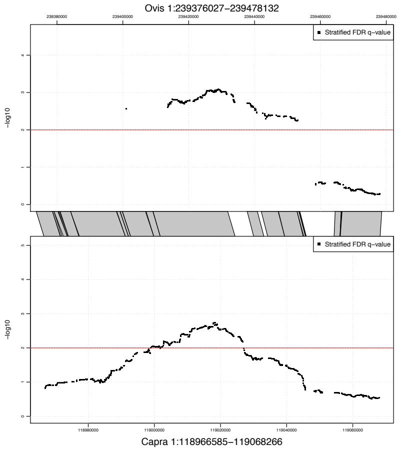

Intergenic

Unknown

Intergenic

Unknown

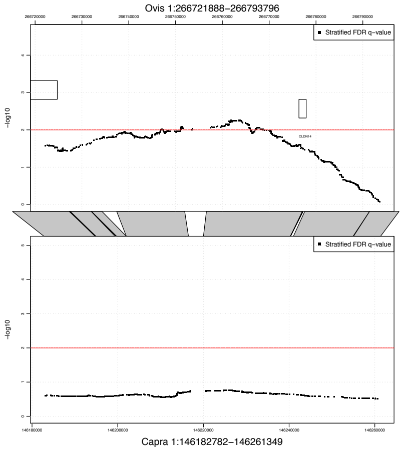

CLDN14

Other (epithelium permeability)

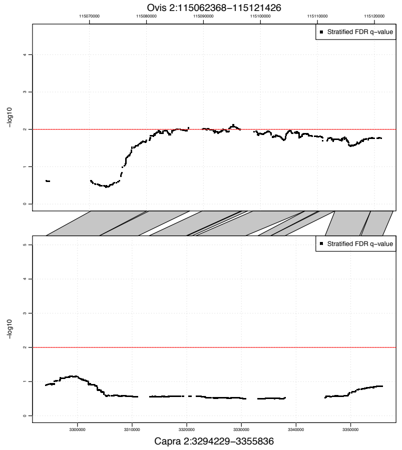

Intergenic

Unknown

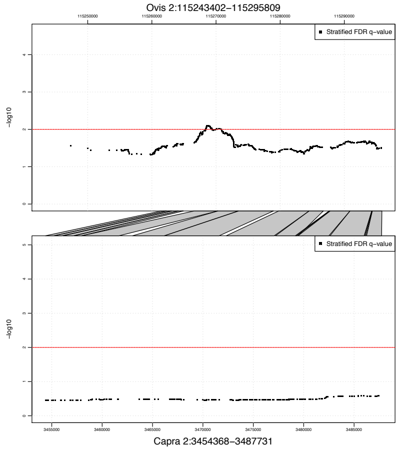

Intergenic

Unknown

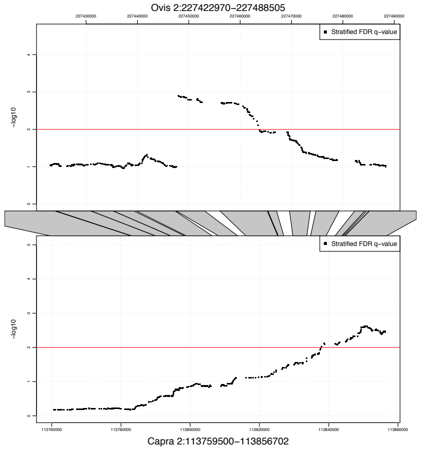

Intergenic

Unknown

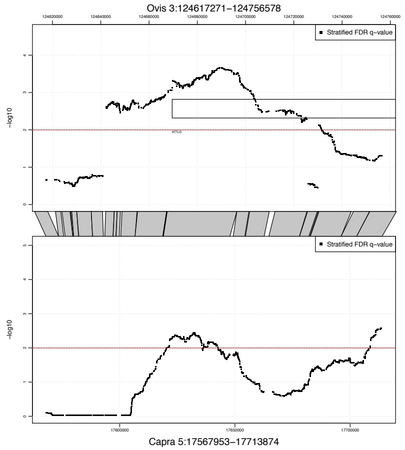

KITLG

Neural stem cell development / Coat colour / Litter size

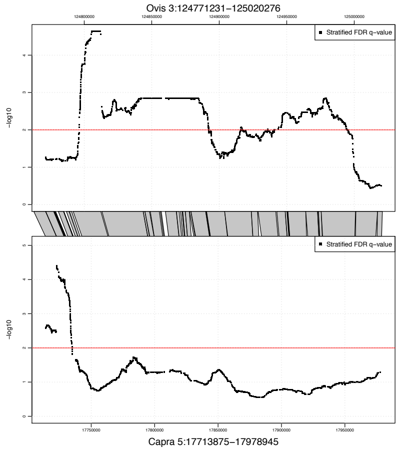

KITLG

Neural stem cell development / Coat colour / Litter size

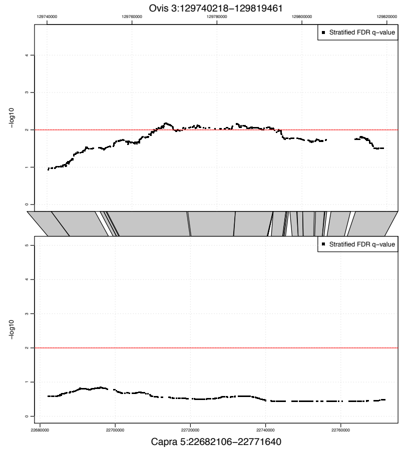

SOCS2

Body weight and growth / Milk production

KITLG

Neural stem cell development / Coat colour / Litter size

Supplementary Fig. 8 continued

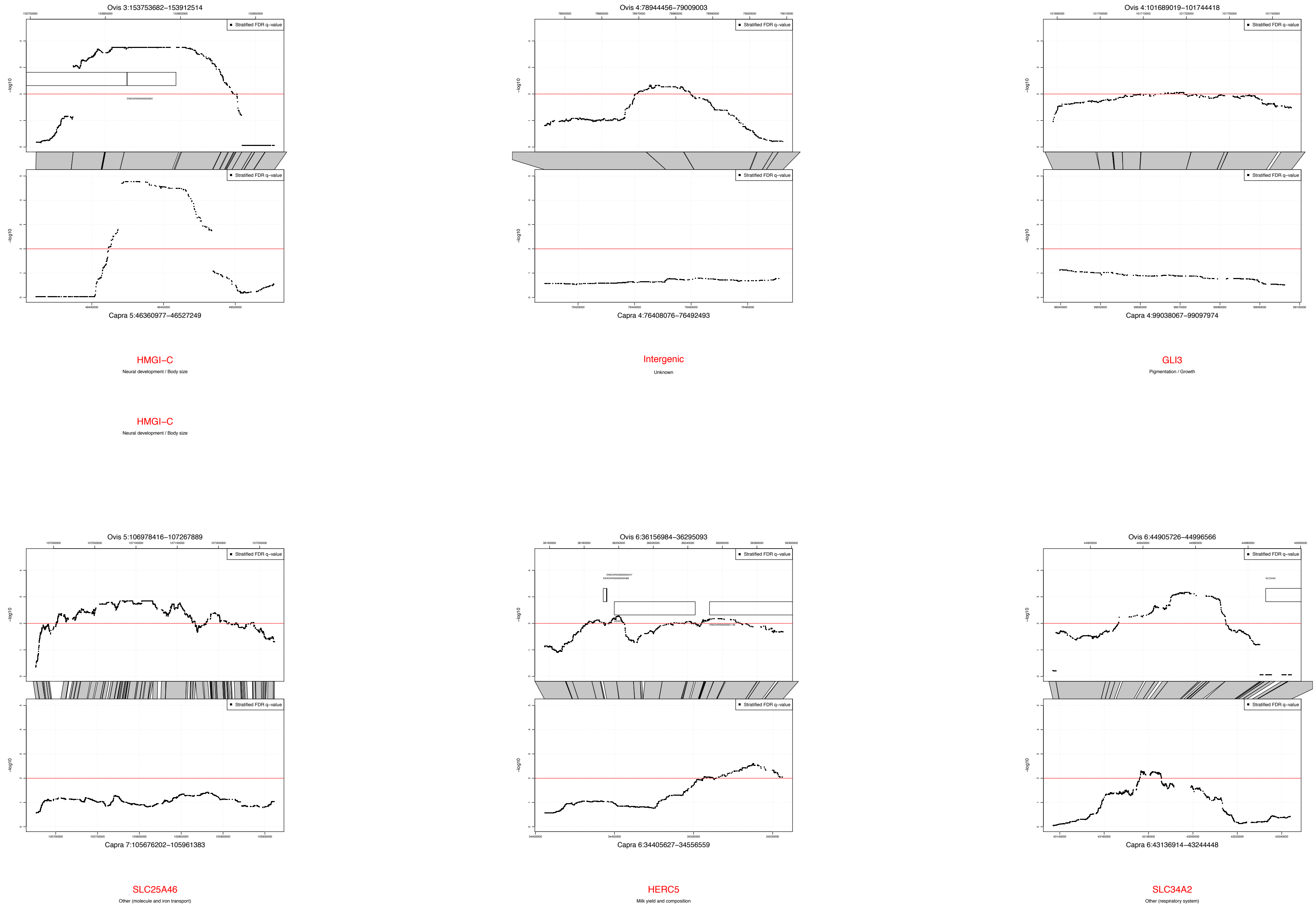

Supplementary Fig. 8 continued

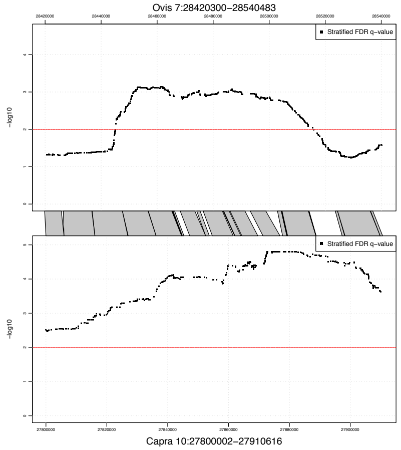

Intergenic

Unknown

Intergenic

Unknown

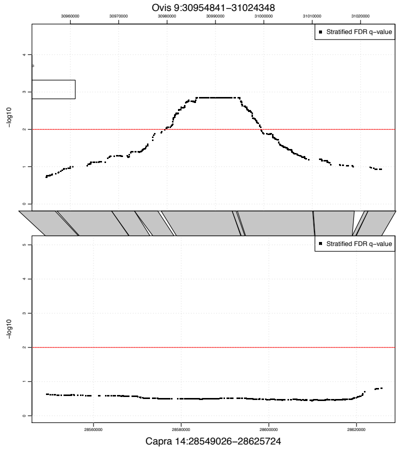

HAS2

Other (extracellular matrix)

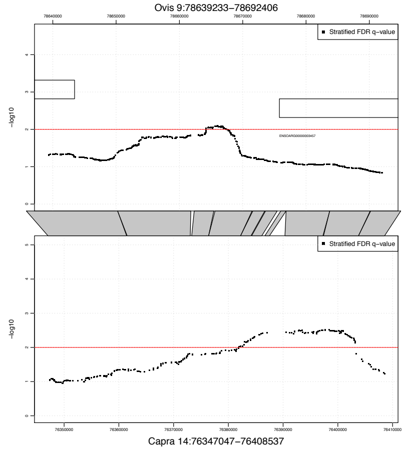

POP1

Other (epithelium development)

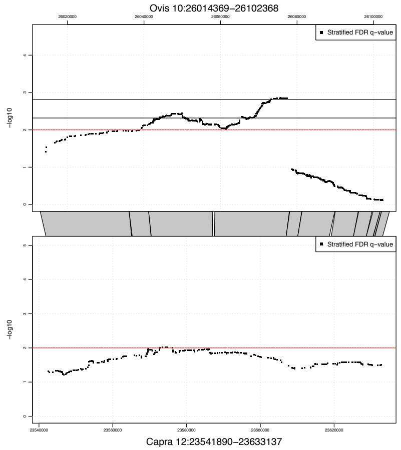

NBEA

Central nervous system / Wool crimp

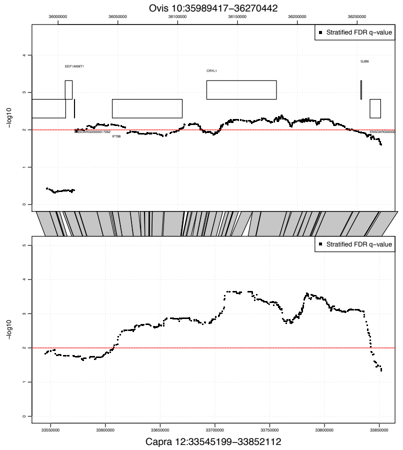

CRYL1

Milk fatty acid content

CRYL1

Milk fatty acid content

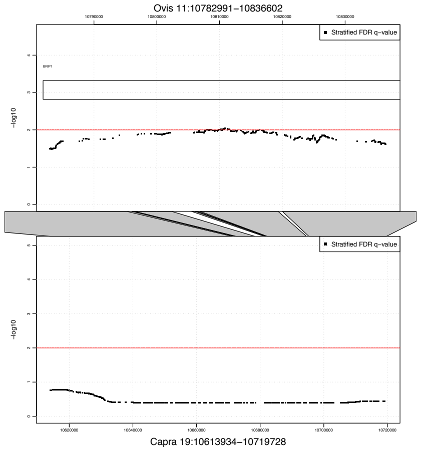

BRIP1

Other (DNA structure)

Supplementary Fig. 8 continued

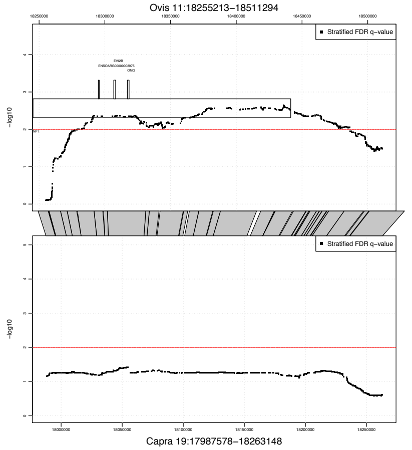

NF1

Neural crest derived tissue development

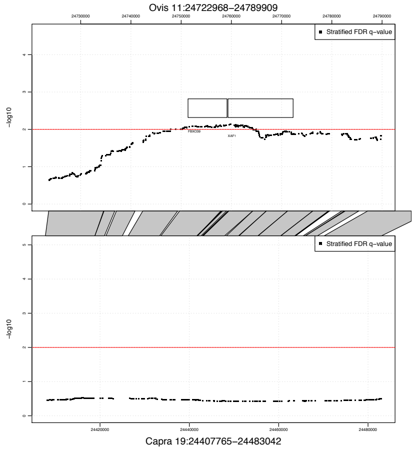

FBXO39

Immunity

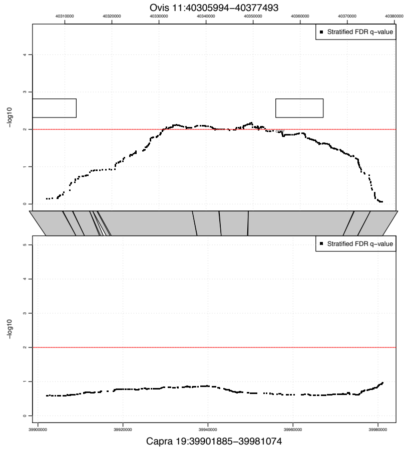

CCR7

Innate immune response

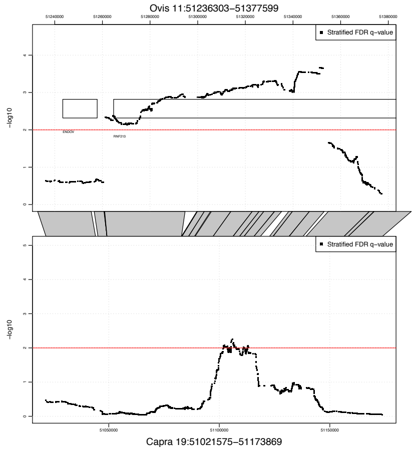

RNF213

Other (angiogenesis)

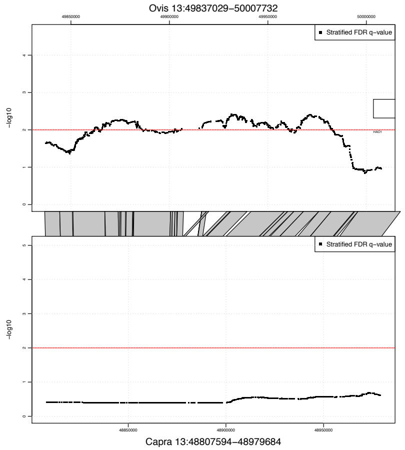

HAO1

Milk content

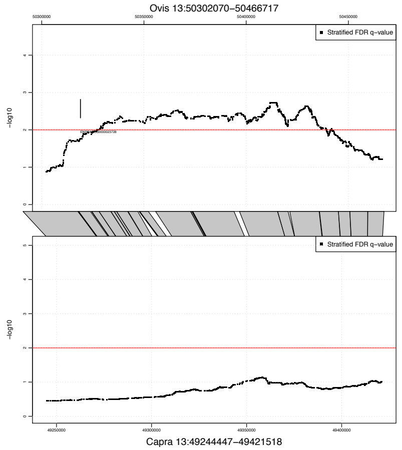

ENSOARG00000023728

Other (Uncharacterised)

Supplementary Fig. 8 continued

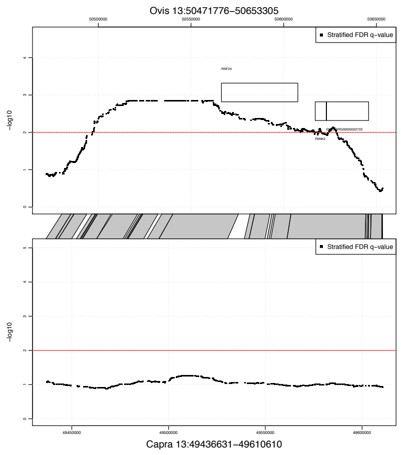

RNF24

Fat tails in sheep

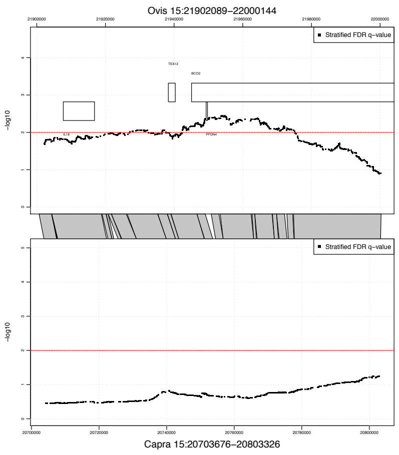

BCO2

Milk and fat colour

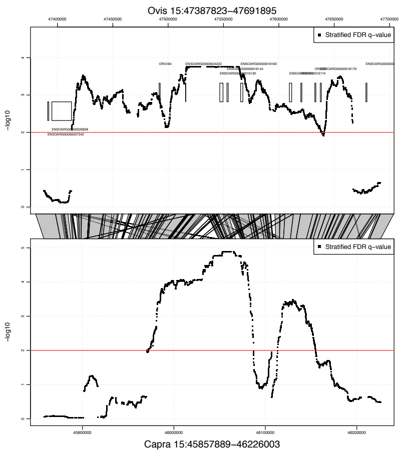

U1

Other (RNA splicing)

HBE1

Other (embryonic metabolism)

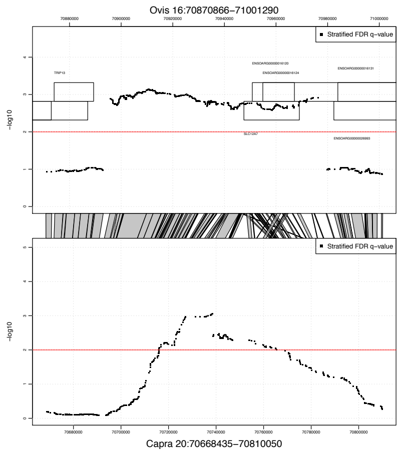

TRIP13

Mastitis susceptibility

SLC12A7

Other (Molecule and ion transport)

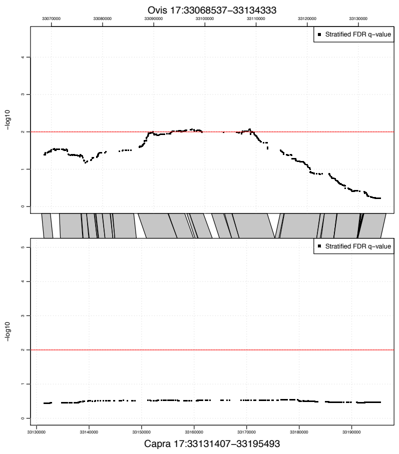

KNTC1

Other (cell division)

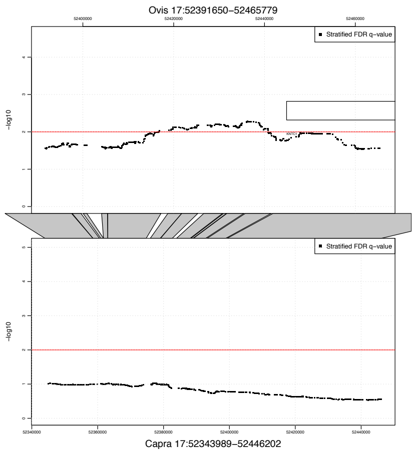

Intergenic

Unknown

Supplementary Fig. 8 continued

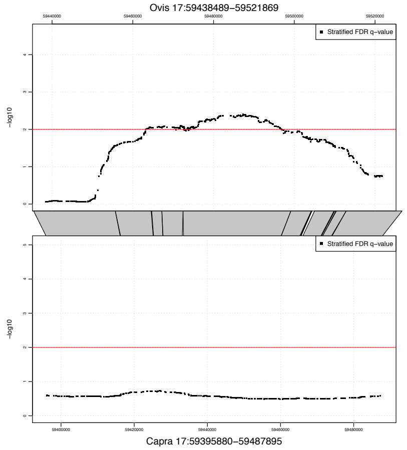

Intergenic

Unknown

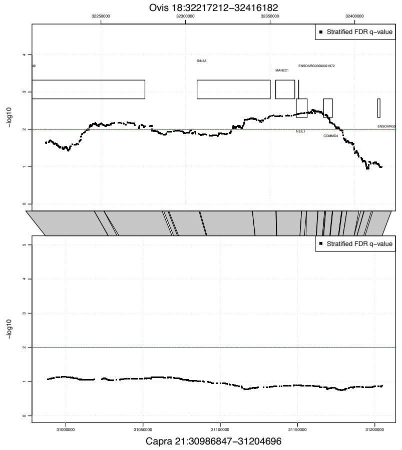

NEIL1

Other (DNA repair pathway)

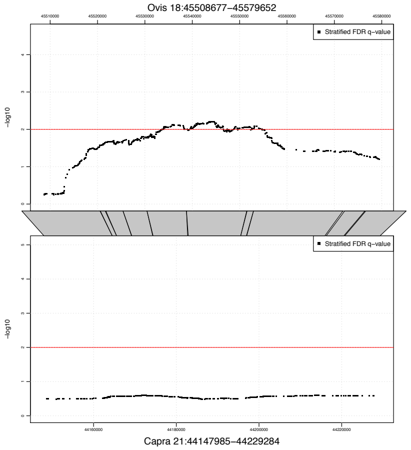

NKX2−1

Fertility

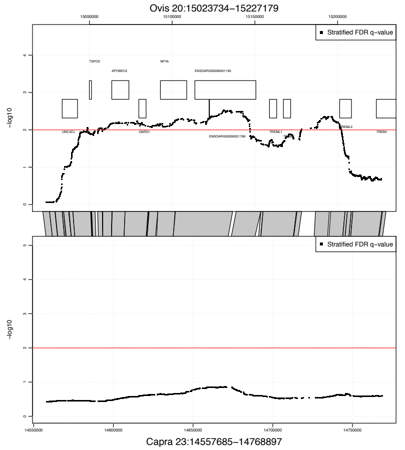

ADCY10

Other (ion binding)

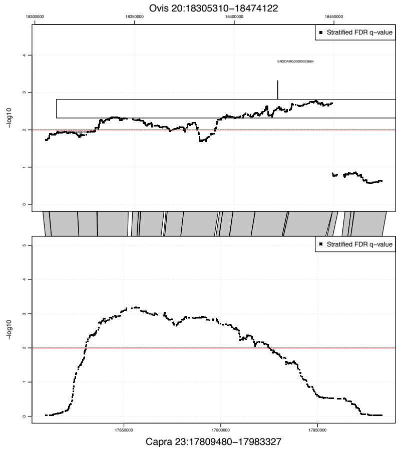

SUPT3H

Milk fatty acid content

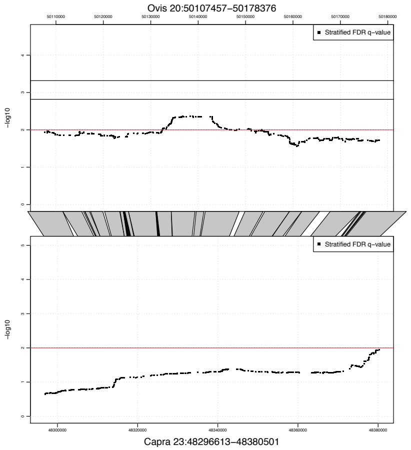

GMDS

Milk fatty acid content

SUPT3H

Milk fatty acid content

Supplementary Fig. 8 continued

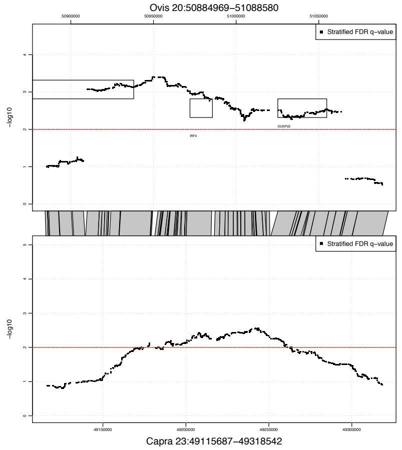

EXOC2

Immunity / Pigmentation

DUSP22

Immunity

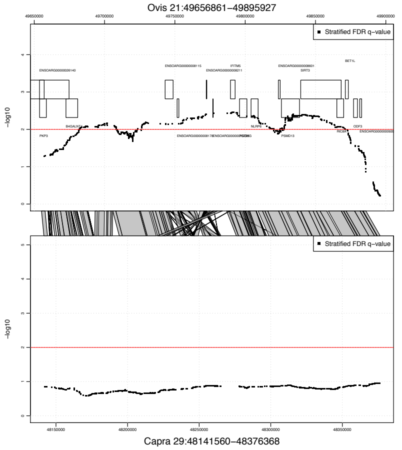

IFITM5

Immunity

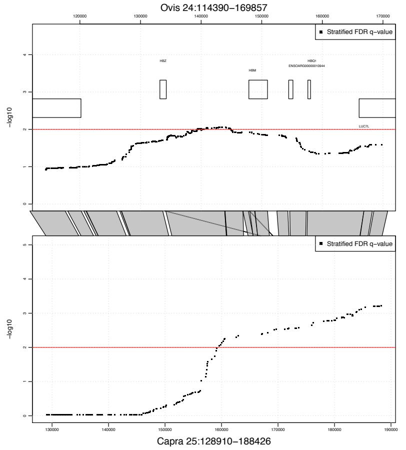

HBM

Other (oxygen transport)

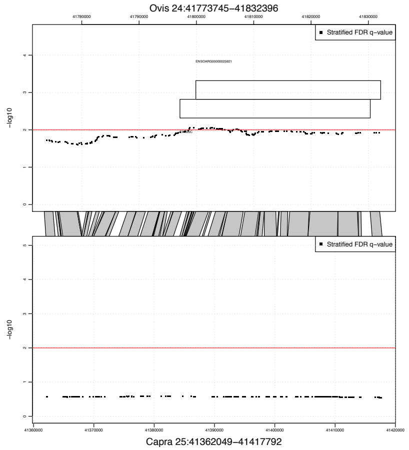

FAM20C

Milk casein composition

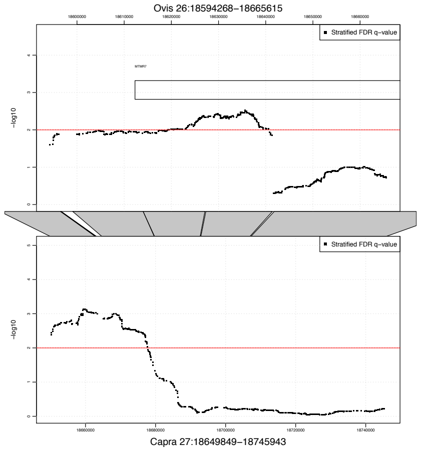

MTMR7

Central nervous system / Muscular fatty acid composition

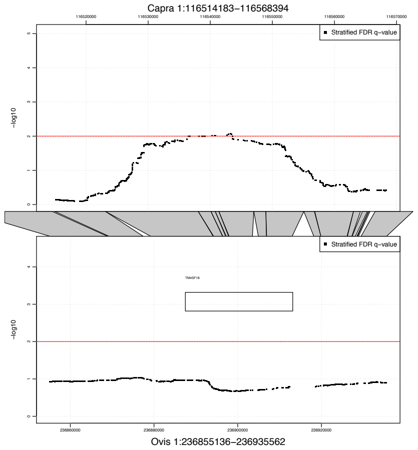

TM4SF18

Immunity

Supplementary Fig. 8 continued

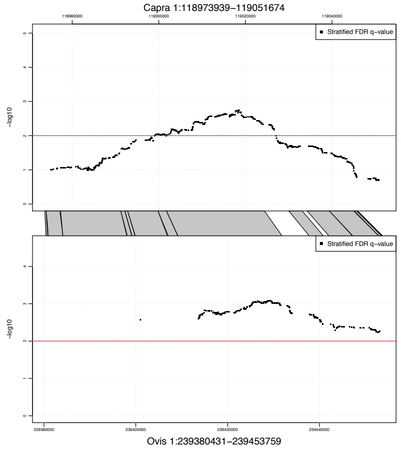

Intergenic
Unknown

Intergenic
Unknown

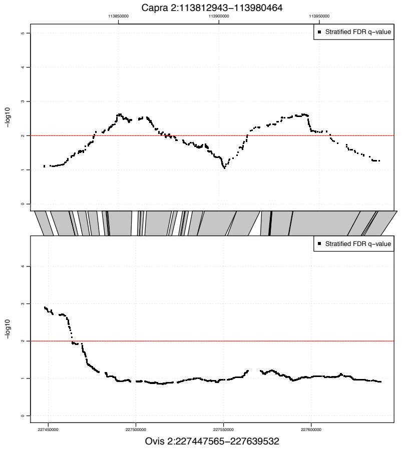

Intergenic
Unknown

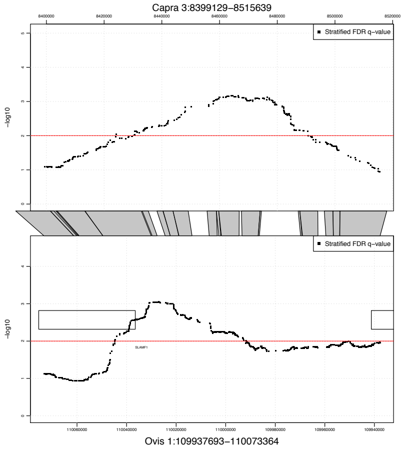

SLAMF1
Immunity

SLAMF1
Immunity

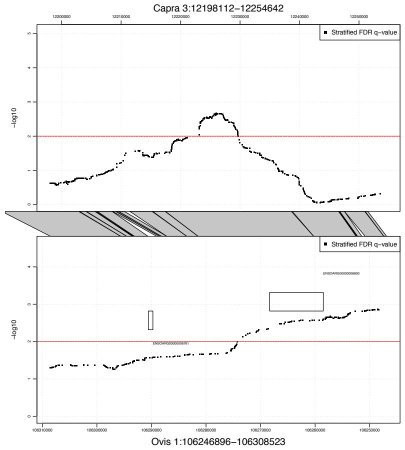

SLAMF8
Immunity

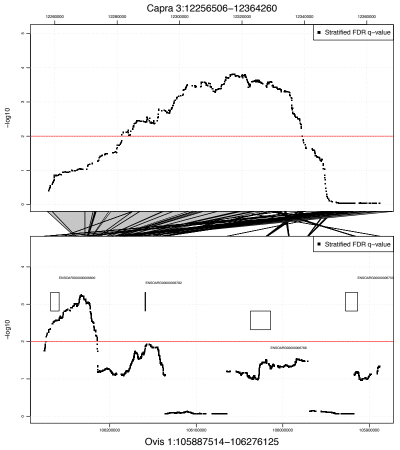

SLAMF7
Immunity

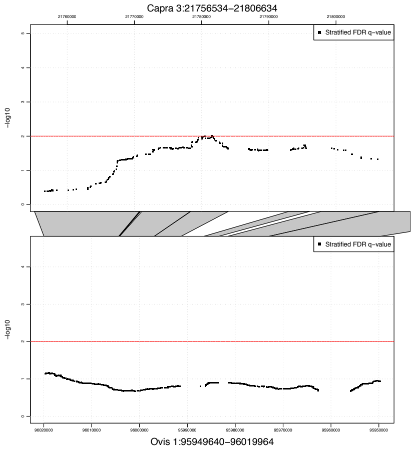

HAO2
Milk content

Supplementary Fig. 8 continued

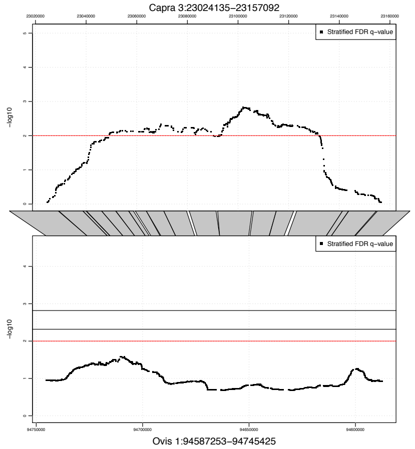

SPAG17

Fatty acid composition

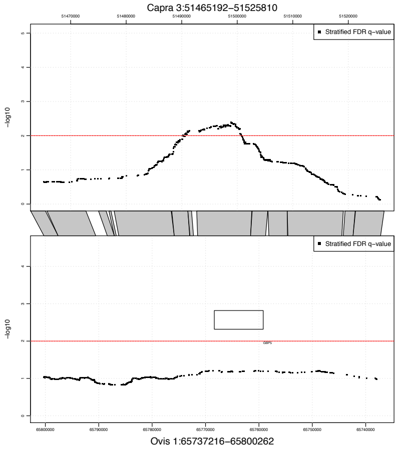

GBP5

Meat quality

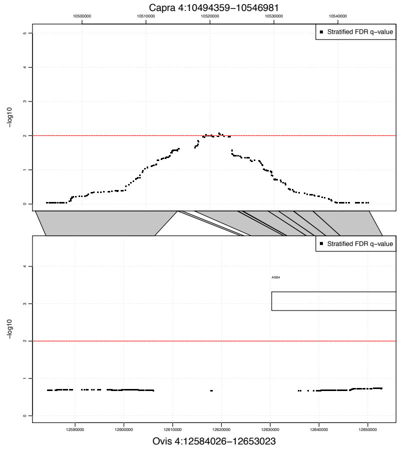

ASB4

Immunity / Vascular development

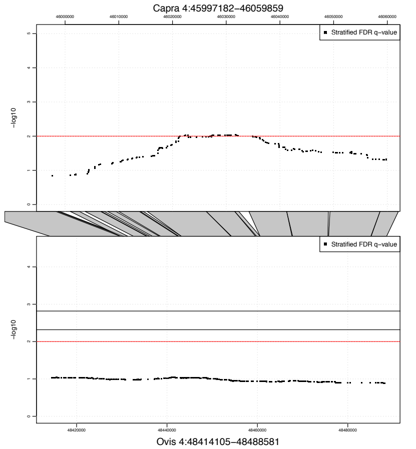

COG5

Fat tails in sheep

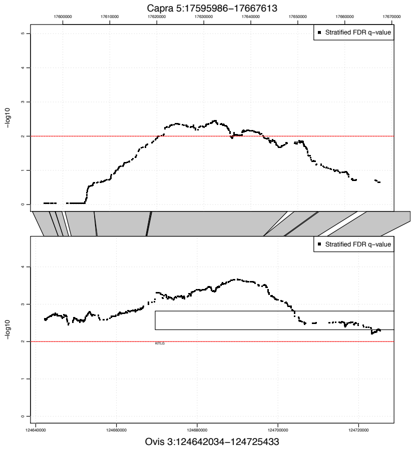

KITLG

Neural stem cell development / Coat colour / Litter size

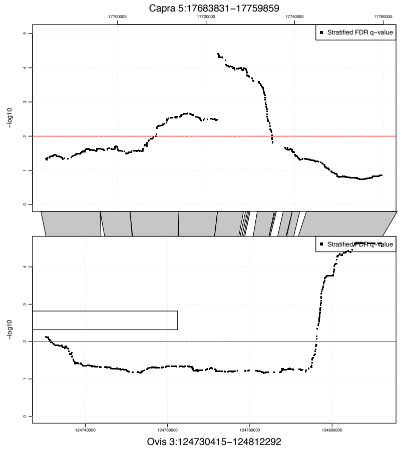

KITLG

Neural stem cell development / Coat colour / Litter size

KITLG

Neural stem cell development / Coat colour / Litter size

Supplementary Fig. 8 continued

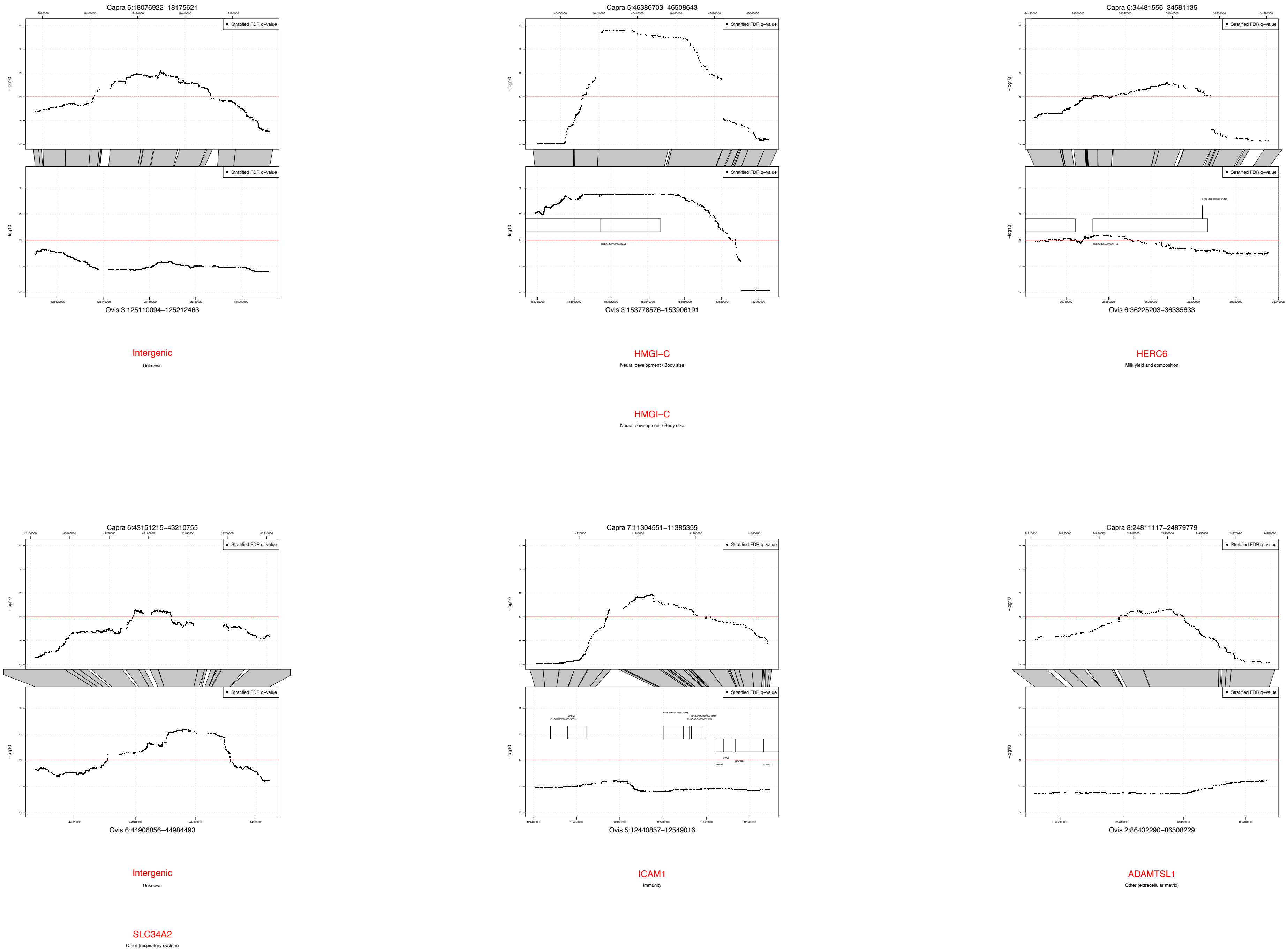

Supplementary Fig. 8 continued

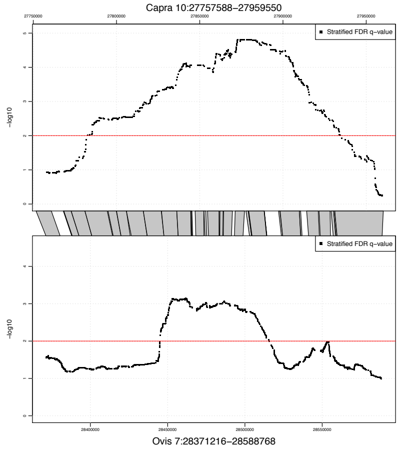

Intergenic

Unknown

Intergenic

Unknown

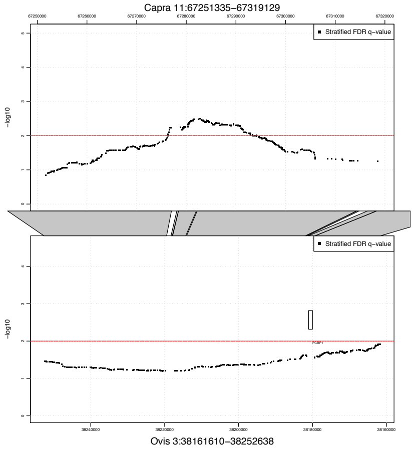

LOC102188412

Other (Uncharacterised)

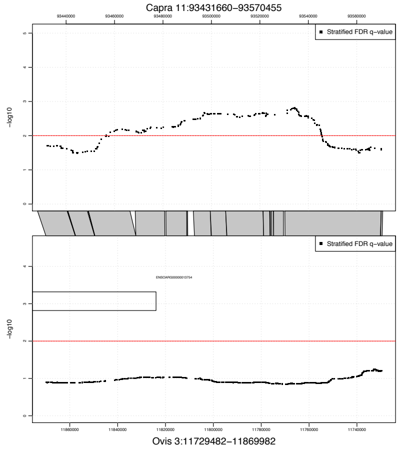

LHX2

Hair follicle development

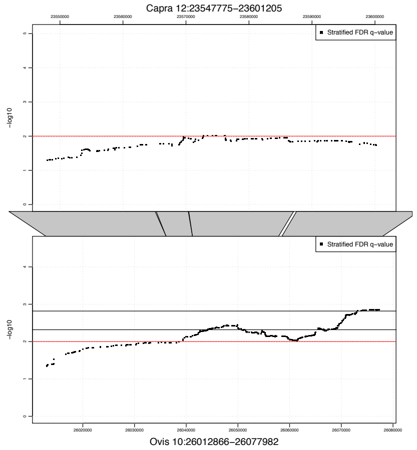

NBEA

Central nervous system / Wool crimp

NBEA

Central nervous system / Wool crimp

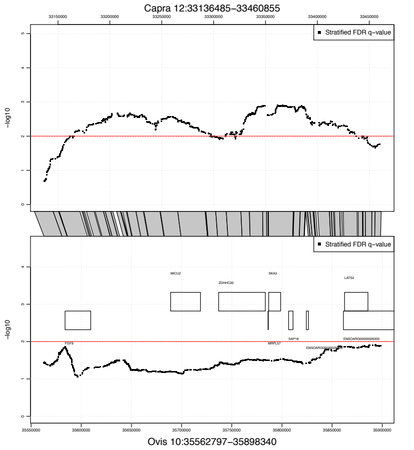

SKA3

Other (cell division)

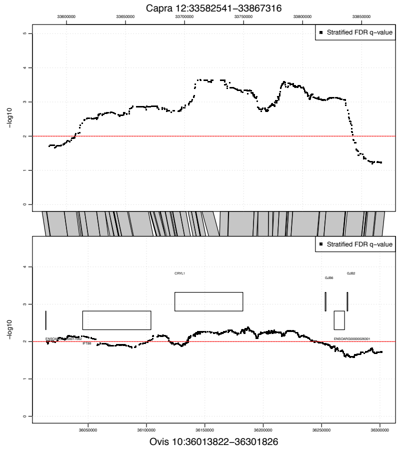

CRYL1

Milk fatty acid content

CRYL1

Milk fatty acid content

Supplementary Fig. 8 continued

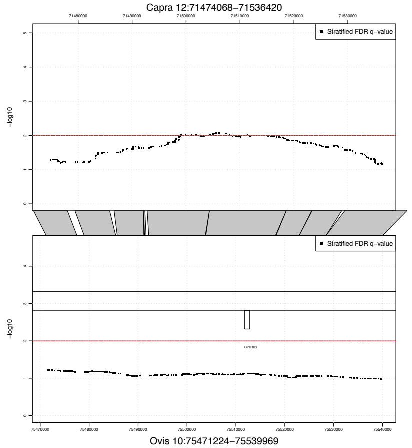

UBAC2
Immunity

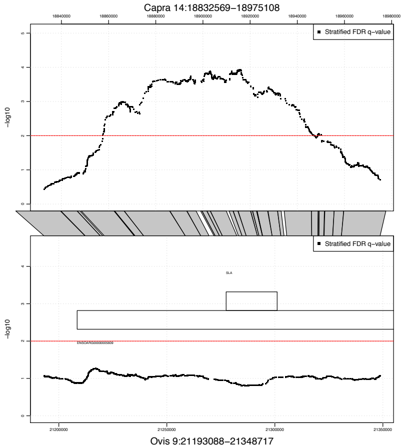

SLA
Immunity

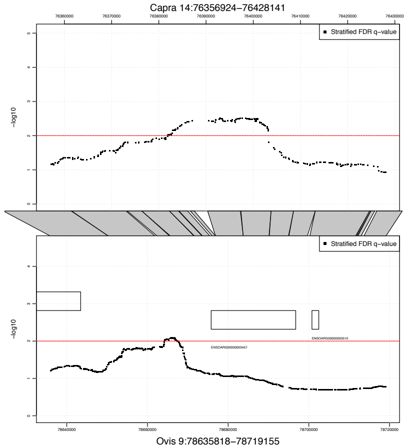

POP1
Other (epithelium development)

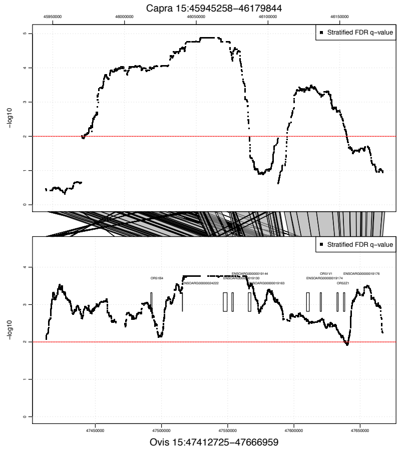

HBE1
Other (embryonic metabolism)

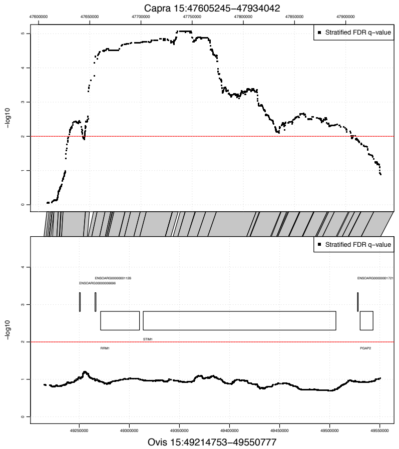

STIM1
Meat quantity

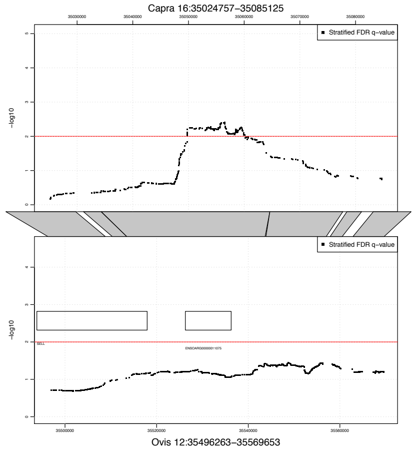

SELE
Immunity

U1
Other (RNA splicing)

Supplementary Fig. 8 continued

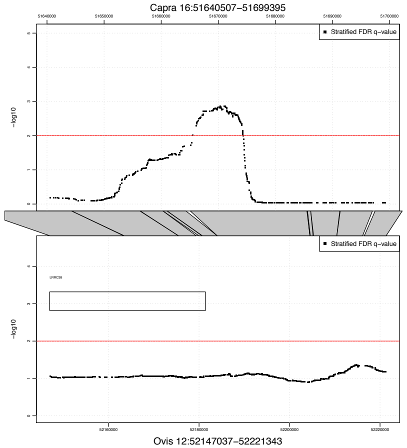

LRRC38

Other (ion transport)

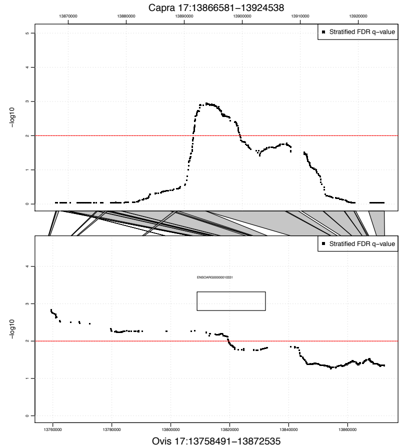

Intergenic

Unknown

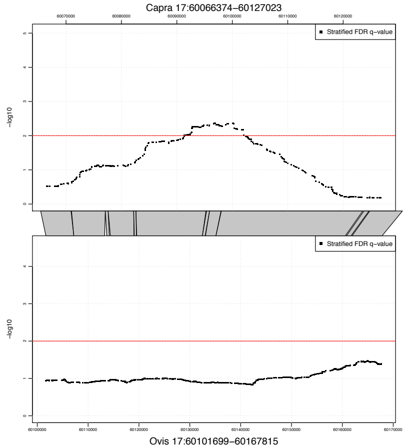

Intergenic

Unknown

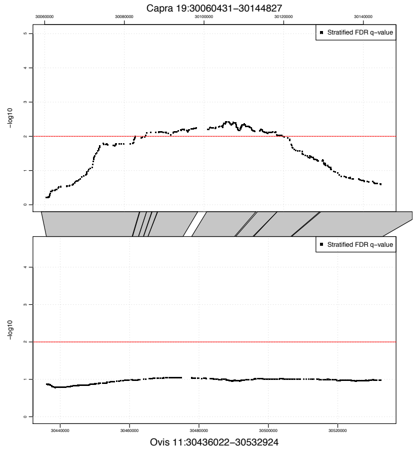

Intergenic

Unknown

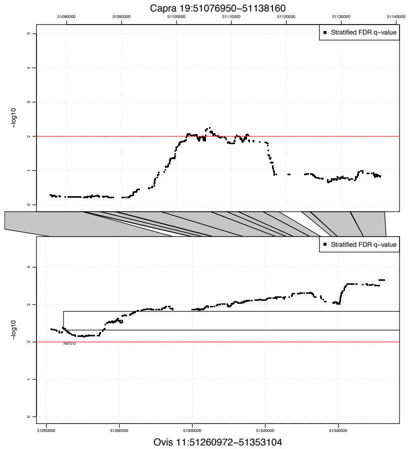

RNF213

Other (angiogenesis)

RNF213

Other (angiogenesis)

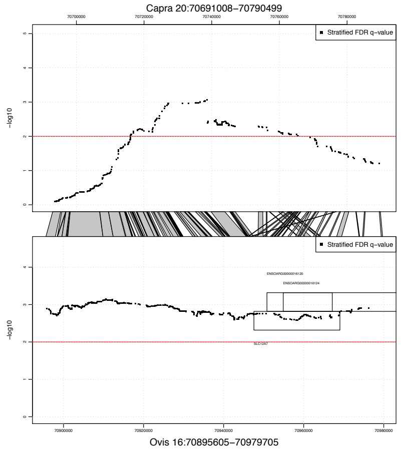

SLC12A7

Other (Molecule and ion transport)

TRIP13

Mastitis susceptibility

Supplementary Fig. 8 continued

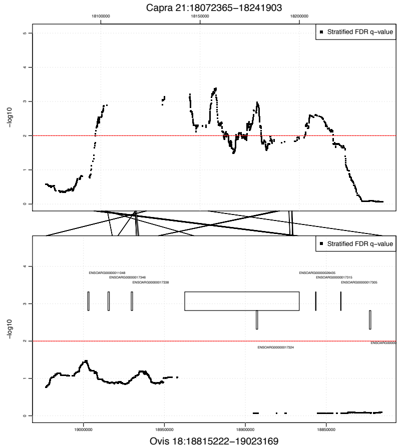

MYADML
Immunity

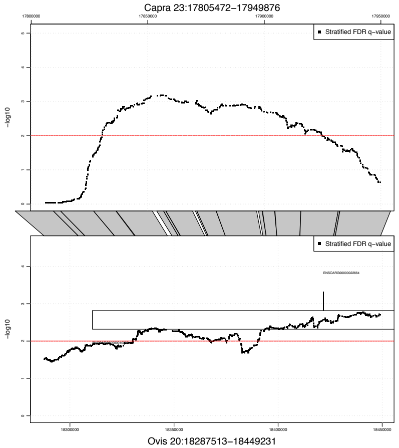

SUPT3H
Milk fatty acid content

SUPT3H
Milk fatty acid content

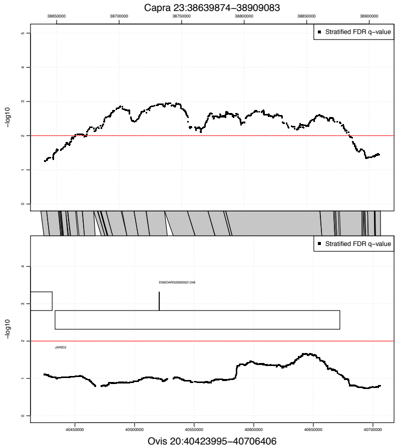

JARID2
Milk protein content

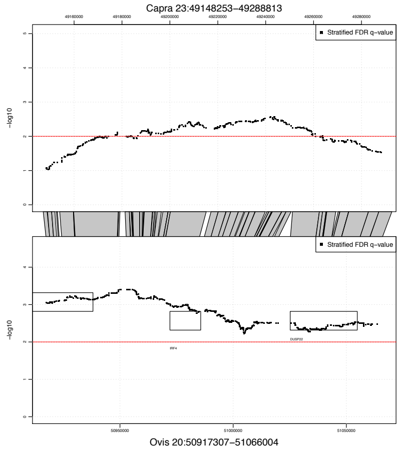

DUSP22
Immunity

EXOC2
Immunity / Pigmentation

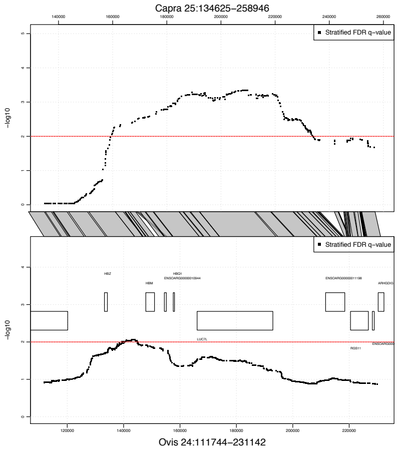

LUC7L
Other (RNA splicing)

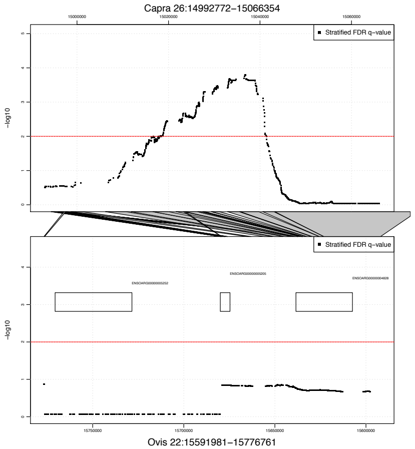

CYP2C19
Other (xenobiotic metabolism)

Supplementary Fig. 8 continued

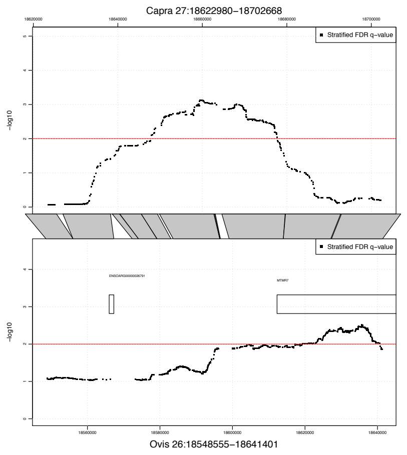

MTMR7

Central nervous system / Muscular fatty acid composition

Supplementary Fig. 8: Combined hapFLK analyses in homologous regions detected under selection in Ovis and/or Capra.

Information is given on position, annotation and function when available for candidate regions. The figure gives the -Log10 of the stratified FDR q-value. The red line represents the q=10-2 threshold.

• **Supplementary Figure 9**

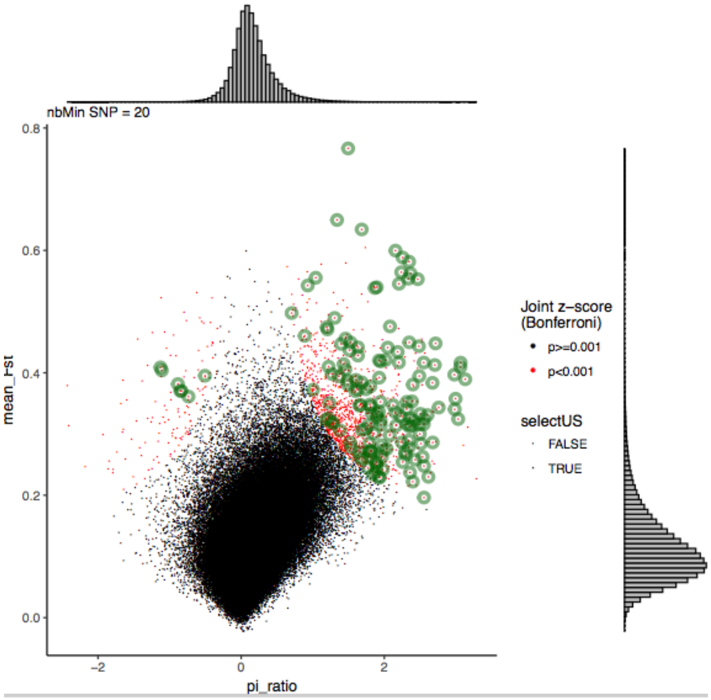

Supplementary Figure 9. Comparison between Fst/pi-ratio and hapFLK approaches for detecting selection.

The figure gives the Fst/ln(pi_ratio) plots for 20kb regions. Of the red dots corresponding to the selective sweeps detected, those within green circles fall into regions detected by hapFLK.

• **Supplementary Figure 10**

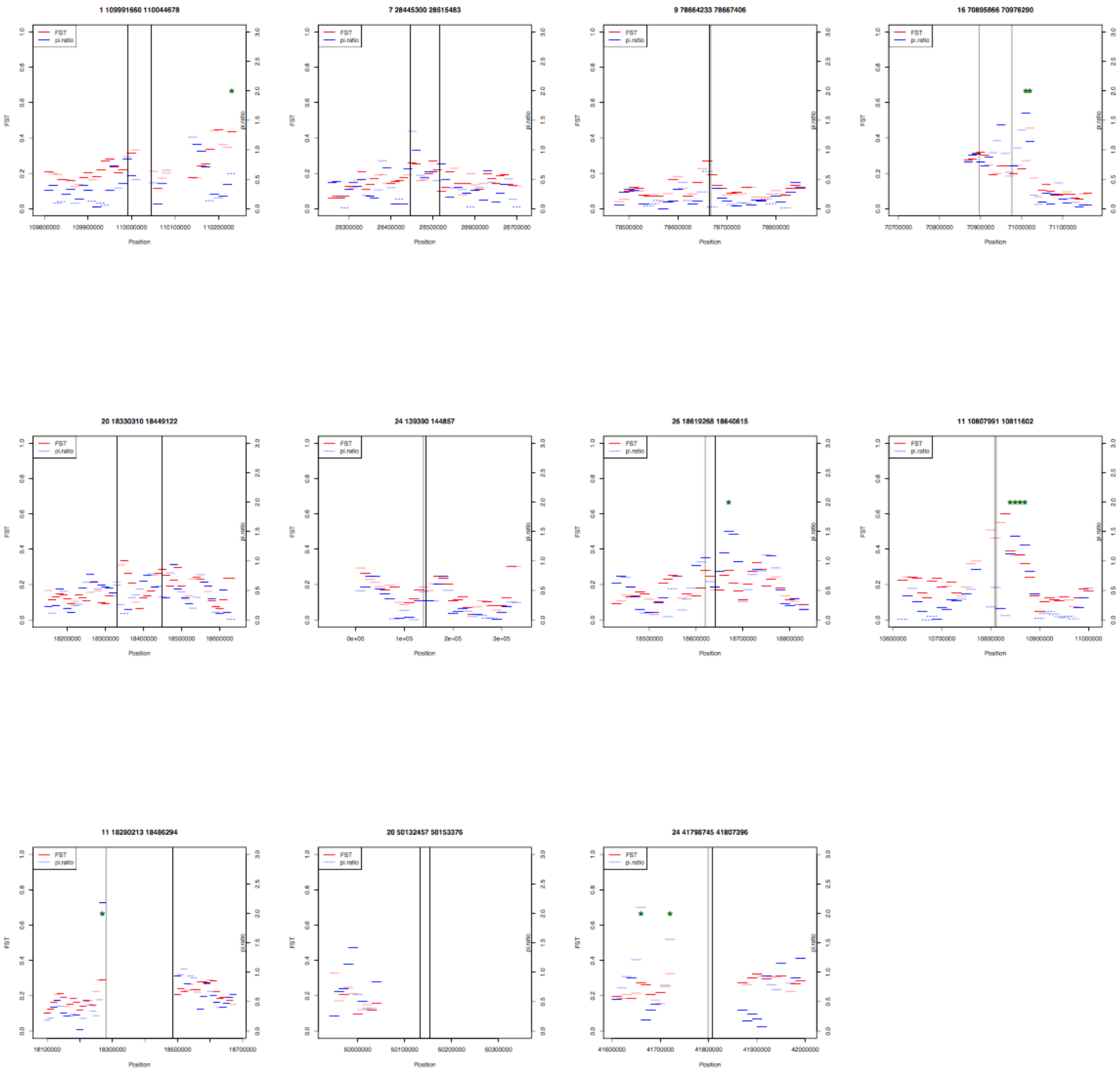

Supplementary Figure 10. Fst (red) and pi_ratio values (blue) along the chromosomes for the 11 regions detected with hapFLK (central demarcated areas) and not with the Fst/ln(pi-ratio) method.

Among them six were close to at least a significant 20 kb region (green stars). Some regions with low SNP densities (< 20 SNPs) were filtered out in the Fst/ln(pi-ratio) method

• **Supplementary Figure 11**

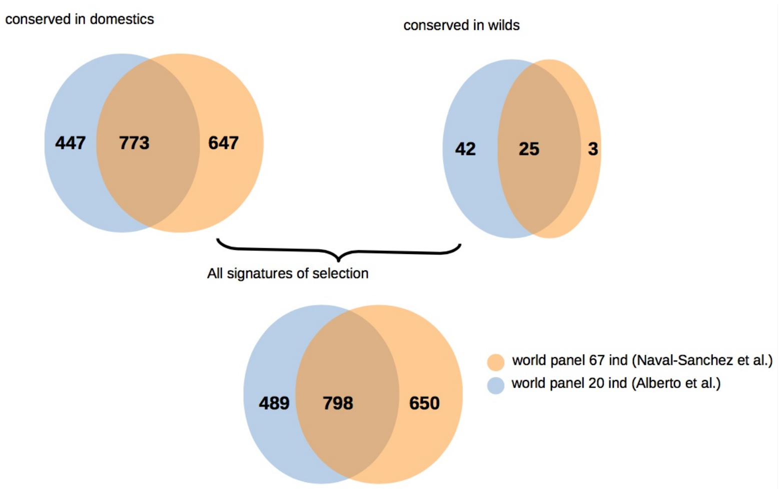

Supplementary Figure 11. Effect of subsampling in the worldwide panel.

The Venn diagrams show the sweeps dtected with the Fst/ln(pi-ratio) method and conserved in domestic animals, wild animals and both according to the world panel chosen. The numbers are for the 20kb-genomic regions detected.

• Supplementary References

1- Naderi, S. et al. The goat domestication process inferred from large-scale mitochondrial DNA analysis of wild and domestic individuals. *Proc. Natl. Acad. Sci. U. S. A.* **105**, 17659–17664 (2008).

2- Frichot, E., Mathieu, F., Trouillon, T., Bouchard, G. & François, O. Fast and efficient estimation of individual ancestry coefficients. *Genetics* **196**, 973–983 (2014).

3- Pickrell, J.K. & Pritchard, J.K. Inference of population splits and mixtures from genome-wide allele frequency data. *PLoS Genetics* https://doi.org/10.1371/journal.pgen.1002967 (2012).

4- Reich, D., Thangaraj, K., Patterson, N., Price, A.L. & Singh, L. Reconstructing Indian population history. *Nature* 461, 489–494 (2009).

5- Larson, G. & Fuller, D. Q. The evolution of animal domestication. *Annu. Rev. Ecol. Evol. Syst*. **45**, 115–136 (2014).

6- Fariello, M.-I. et al. Selection signatures in worldwide sheep populations. *PLoS One* https://doi.org/10.1371/journal.pone.0103813 (2014).

7- Ruyter-Spira, C.P. et al. The HMGI-C gene is a likely candidate for the autosomal dwarf locus in the chicken. *Journal of Heredity* **89**, 295–300 (1998).

8- Sanchez-Juan, P. et al. Genome-wide study links MTMR7 gene to variant Creutzfeldt-Jakob risk. *Neurobiology of Aging* **33**, 1487.e21–1487.e28 (2012).

9- Ramayo-Caldas, Y. et al. Liver transcriptome profile in pigs with extreme phenotypes of intramuscular fatty acid composition. *BMC Genomics* https://doi.org/10.1186/1471-2164-13-547 (2012).

10- Charon, K.M., Lipka, K.R. The effect of a coat colour-associated genes polymorphism on animal health–a review. *Annals of Animal Science* **15**, 3–17 (2015).

11- An, X.P. et al. Two mutations in the 5′-flanking region of the KITLG gene are associated with litter size of dairy goats. *Animal Genetics* **46**, 308–311(2015).

12- Scherer, D. & Kumar, R. Genetics of pigmentation in skin cancer—a review. *Mutation Research/Reviews in Mutation Research* **705**, 141–153 (2010).

13- Wang, Z. et al. Genome-wide association study for wool production traits in a Chinese Merino sheep population. *PLoS One* https://doi.org/10.1371/journal.pone.0107101 (2014).

14- Nair, R. et al. Neurobeachin regulates neurotransmitter receptor trafficking to synapses. *Journal of Cell Biology* **200**, 61–80 (2012).

15- Ramey, H.R. et al. Detection of selective sweeps in cattle using genome-wide SNP data. *BMC Genomics* https://doi.org/10.1186/1471-2164-14-382 (2013).

16- Naval-Sanchez, M. et al. Sheep genome functional annotation reveals proximal regulatory elements contributed to the evolution of modern breeds. *Nat. Commun*. (in press).
