# Supplementary material for: Convergent genomic signatures of domestication in sheep and goats
Source: Nat Commun. 2018 Mar 6;9:813. doi: 10.1038/s41467-018-03206-y (PMC5840369; doi:10.1038/s41467-018-03206-y)
Supplement: Supplementary file 3 — Description of Additional Supplementary Files [file 41467_2018_3206_MOESM3_ESM.pdf]

**Description of Additional Supplementary Files**

File Name: Supplementary Data 1
Description: Mean and individual genetic load for genes showing higher average load in sheep than in Asiatic mouflon.

File Name: Supplementary Data 2
Description: Results of the three populations tests for *Ovis* and *Capra*.

File Name: Supplementary Data 3
Description: Genomic regions under selection with the corresponding genes and functions in *Ovis* and *Capra*.

File Name: Supplementary Data 4
Description: Functions related to genes under selection in regions either common or specific to *Capra* and *Ovis*.

File Name: Supplementary Data 5
Description: Origin of the *Ovis* and *Capra* samples, with individual values for inbreeding and genetic load.
